# The Zuo1 C-terminal domain stabilizes DNA guanosine quadruplex (G4) structures located on Chromosome IX in *Saccharomyces cerevisiae*

**DOI:** 10.1093/nar/gkaf1055

**Published:** 2025-11-03

**Authors:** Ines Burkhart, Michaela Limmer, J Carlos Penedo, Li-Chia Sauer, Harald Schwalbe, Katrin Paeschke

**Affiliations:** Institute for Organic Chemistry and Chemical Biology, Center for Biomolecular Magnetic Resonance, Goethe-University Frankfurt, Max-von-Laue-Str. 7, 60438 Frankfurt/Main, Germany; Department of Oncology, Hematology and Rheumatology, University Hospital Bonn, 53127 Bonn, Germany; Department of Clinical Chemistry and Clinical Pharmacology, University Hospital Bonn, 53127 Bonn, Germany; Centre of Biophotonics, Laboratory for Biophysics and Biomolecular Dynamics, School of Physics and Astronomy, University of St Andrews, North Haugh, St Andrews KY16 9SS, United Kingdom; School of Biology, University of St Andrews, North Haugh, St Andrews KY16 9ST, United Kingdom; Department of Clinical Chemistry and Clinical Pharmacology, University Hospital Bonn, 53127 Bonn, Germany; Institute for Organic Chemistry and Chemical Biology, Center for Biomolecular Magnetic Resonance, Goethe-University Frankfurt, Max-von-Laue-Str. 7, 60438 Frankfurt/Main, Germany; Department of Oncology, Hematology and Rheumatology, University Hospital Bonn, 53127 Bonn, Germany; Department of Clinical Chemistry and Clinical Pharmacology, University Hospital Bonn, 53127 Bonn, Germany

## Abstract

Deoxyguanosine quadruplexes (G4s) form stable non-B-DNA structures that can affect transcription, replication, and genome stability. Depending on various factors including cation binding, G4s can fold into different topologies, which can be linked to distinct function. In cells, G4 folding, function, and unfolding is affected by proteins that specifically target G4s. Zuo1 is a G4-binding protein in yeast. To investigate Zuo1 binding and its consequences on G4 formation and topology, we characterized Zuo1’s interaction with G4s, both *in vitro* and *in vivo*. The C-terminus (Zuo1_348-433_) of Zuo1 interacts with the G4s. We characterized this interaction by combining nuclear magnetic resonance spectroscopy, single-molecule Förster Resonance Energy Transfer (smFRET), and *in vivo* experiments with G4_IX_ that is located on yeast chromosome IX. The Zuo1–G4_IX_ interaction stabilizes this G4 structure and triggers conformational shifts depending on the cation environment. The data presented here demonstrate that Zuo1 targets a specific conformation state of G4 IX, modulates G4 toppology.

## Graphical Abstract

**Graphical Abstract:**
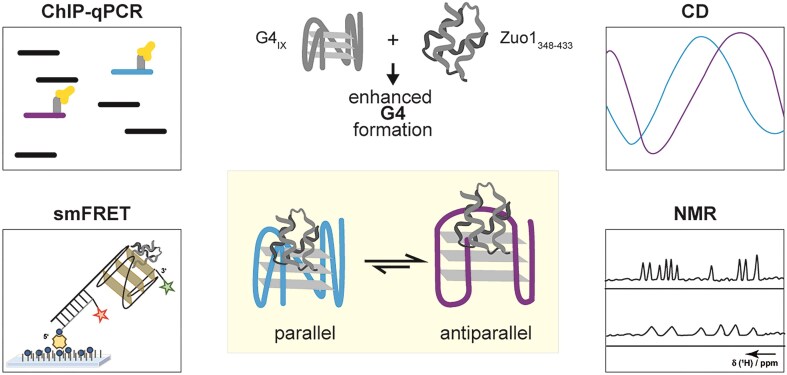

## Introduction

For a long time, DNA function was exclusively linked to its B-form structure, with its role limited to coding for genes. However, it is now well documented that non-B-DNA structures can affect cellular function directly including DNA replication, nucleosome binding, gene expression, and DNA recombination [1, 2]. Non-B-DNA structures include the left-handed Z-DNA, cruciform, intramolecular triplexes, slipped-strand DNA, parallel-stranded DNA, unpaired DNA strands, and notably Deoxyguanosine quadruplexe (G4) DNA structures [3–7]. Among these, G4s stand out as functionally relevant DNA structures that fold both *in vitro* and *in vivo* across all tested organisms [8, 9].

DNA G4 structures require specific deoxyguanosine-rich motifs that contain four deoxyguanosine tracts (G-tracts) separated by regions of variable nucleotide composition. The deoxyguanosines in the G-tracts form Hoogsteen hydrogen bonds with deoxyguanosines in adjacent G-tracts, leading to a cyclic arrangement with four deoxyguanosines called a G-quartet. Stacking of these G-quartets results in the 3D structure known as the G4. Initially, the consensus G4 motif was defined as (G ≥ 3)N_1–7_(G ≥ 3)N_1–7_(G ≥ 3)N_1–7_(G ≥ 3), where N represents variable regions that form loops in the 3D structure [10]. Both the G-tracts and the loop regions affect the stability of the G4 structures. A larger number of deoxyguanosines in each of the four G-tracts, as well as shorter loops, increase the stability of G4s [11–13]. Although it was long assumed that G4s could only fold if they harbored strictly the consensus G4 motif, various experiments have demonstrated, both *in vitro* and *in vivo*, that G4 structures can also form within regions containing nonconsensus motifs that have only two guanines per G-tract and/or longer, variable loop regions [14]. As discussed in the literature [9], nonconsensus G4s are predicted to have reduced stability and are therefore classified as metastable [15]. Given their sequence requirements, it is noteworthy that G4s are polymorphic, and even a single G4 sequence can adopt different topologies (e.g. parallel, antiparallel, or hybrid) [16–18]. Further, G4 structures incorporating nonconsensus motifs can form additional structures that include bulges [12, 19], strand interruptions with snapback deoxyguanosines [20] and incomplete tetrads [21, 22]. It has remained elusive how different G4 topologies form and which impact these have on G4-related cellular functions.

So far, consensus and nonconsensus G4 motifs were detected in the genomes of all tested organisms [14, 23]. In human, for example, over 50% of the 700 000 potential G4 motifs have nonconsensus G4 motifs. In *Saccharomyces cerevisiae*, over 500 G4 motifs were identified [24] and most of those have nonconsensus G4 motifs [23]. Experiments in yeast demonstrated that nonconsensus G4 motifs can form stable structures, which can modulate transcription and impact genome instability, if not regulated by helicases [25–28]. The current model suggests that G4s form transiently within cells, and that their folding, unfolding, and stability are regulated by proteins that both enable G4 function and prevent genome instability [29–32].

Previously, we have identified Zuo1 as a G4-binding protein in *S. cerevisiae* [33]. Even though Zuo1 is a component of the ribosome-associated complex (RAC) [34], it is also located in the nucleus and functions in the regulation of transcription [35]. We demonstrated that Zuo1 binds to DNA G4 structures *in vitro* and and associates with G4 motifs in cells, as shown by ChIP-seq [33]. Zuo1-deficient (*zuo1Δ*) yeast cells exhibit fewer folded G4s in the nucleus. *zuo1Δ* cells showed defects in nucleotide excision repair (NER) after ultraviolet (UV) irradiation [33]. These findings indicate that Zuo1 may stabilize G4s or even induce their formation, both of which are needed for correct DNA repair following UV-induced damage [33]. Similar to yeast, the human orthologue of Zuo1, ZRF1, binds to G4 motifs and affects cellular responses after UV irradiation [36].

Here, we investigate whether and to which extent Zuo1 stabilizes or induces the formation of G4 structures. Our investigations build on previous findings showing that Zuo1 binds to a G4 on chromosome IX in yeast (G4_IX_). According to the definition by Marsico *et al.* [23], G4_IX_ has not a strict consensus G4 motif, as the loop length is extended; it is therefore classified as nonconsensus. We reveal that the C-terminal region of Zuo1 binds the nonconsensus G4_IX_ motif and influences both its formation and stability. Our findings are the first to demonstrate that a specific protein can bind to, stabilize, and promote the formation of a nonconsensus, less stable G4 structure.

Nonconsensus G4s are prevalent and overrepresented across multiple genomes. Their biological relevance has been debated, as their inherent instability may limit their ability to regulate cellular processes. Our work supports the hypothesis that nonconsensus G4s can be stabilized by specific proteins, rendering them functional and capable of influencing cell behavior as regulatory elements.

## Materials and methods

### Strains, constructs, and media

All yeast strains are listed in Supplementary Table S1. All experimental strains are derivatives of the *RAD5 +* version of W303 (R. Rothstein) [37]. Deletions eliminated entire ORFs and were created according to [37]. Epitope tagging was carried out as described [38]. Myc-tagged proteins were expressed from endogenous loci and promoters.

### Oligonucleotides

For circular dichroism (CD) and nuclear magnetic resonance (NMR) experiments, the oligonucleotides listed in Supplementary Table S2 and a G4 structure located on the chromosome IX of *S. cerevisiae* (G4_IX_; 5′-GGGTACGGTGGGTAATAAGGGAAGGTATCGGG-3′) were used, as well as the mutated version (G4_IXmut_; 5′-GCGTACGGTGCGTAATAAGCGAAGGTATCGCG-3′). For microscale thermophoresis (MST) measurements, a Cy5-label was added to the 5′-end of the respective DNAs. 5′-TGGCGACGGCAGCGACCATTTGGGTACGGTGGGTAATAAGGGAAGGTATCGGG-Cy3-3′ and 5′-Cy5-TGGTCGCTGCCGTCGCCA-Biotin-3′ oligonucleotides were used for single-molecule FRET (smFRET) experiments (the G4_IX_ sequence is underlined).

### CD spectroscopy

CD spectra were recorded on a Jasco J-810 spectropolarimeter in a 2 mm quartz glass cuvette at 25°C using 7.5 µM DNA for each measurement. The samples were measured in K^+^ buffer (25 mM potassium phosphate, pH 7.0) or Na^+^ buffer (100 mM NaCl, 10 mM Tris∙HCl, pH 7.0) in a range from 200 to 320 nm. All curves were smoothed by a Savitzky–Golay function. For the *in situ* experiments 1-5 eq protein were injected into 7.5 µM DNA during each measurement. This was done manually as part of a homebuilt mixing set-up. Kinetic traces were obtained at single wavelengths of interest (265 and 295 nm) and monitored over time. The resulting data were fitted with a mono- or a bi-exponential function:


\begin{eqnarray*}
{\mathrm{f(x) = a}} \cdot {\mathrm{(1 - }}{{\mathrm{e}}}^{{\mathrm{( - b}} \cdot {\mathrm{x)}}}{\mathrm{)}}
\end{eqnarray*}

\begin{eqnarray*}
{\mathrm{f(x) = a}} \cdot {\mathrm{(1 - }}{{\mathrm{e}}}^{{\mathrm{( - b}} \cdot {\mathrm{x)}}}{\mathrm{) + c}} \cdot {\mathrm{(1 - }}{{\mathrm{e}}}^{\left( { - {\mathrm{d}} \cdot {\mathrm{x}}} \right)}{\mathrm{)}}
\end{eqnarray*}

Thermal melting was performed from 5°C or 25°C to 95°C with a heating rate of 0.5°C min^−1^ and recorded at the absorption maximum of the respective CD trace. Melting curves were fitted with a sigmoidal function. List of used oligonucleotides are in Supplementary Table S2.

### Cloning, expression, and purification of Zuo1

Zuo1_348–433_ and Zuo1 full length were cloned with *Nde*I and *Bam*HI as restriction sites into a pET-TEV vector (GenScript). Correct cloning was confirmed by sequencing. Zuo1_348–433_ was expressed as a fusion protein in T7 express competent *E. coli* cells (New England BioLabs) carrying a N-terminal His_6_-tag and a TEV (tobacco etch virus) cleavage site. For uniform ^15^N-labeling, cells were grown in M9 medium supplemented with 1 g l^-1 15^NH_4_Cl and 100 µg ml^−1^ ampicillin. Expression was induced with 1 mM isopropyl β-D-thiogalactoside at OD_600_ = 0.6 and carried out at 37°C for 3 h. Cells were harvested at 4000 × *g* and 4°C for 30 min. Cell lysis was performed in lysis buffer (300 mM NaCl, 20 mM HEPES, pH 7.0, 1 mM 1,4-Dithiothreitol (DTT), 5 mM imidazole) supplemented with a cOmplete^TM^ protease-inhibitor tablet (Roche, Germany) using a french press Microfluidics M-100P at a pressure of 15 000 PSI (pounds per square inch) under continuous cooling to 0°C. The supernatant of centrifuged cell lysate was applied onto a 5 ml HisTrap HP column (GE Healthcare) pre-equilibrated with lysis buffer. Bound protein was eluted with elution buffer (300 mM NaCl, 20 mM HEPES, pH 7.0, 1 mM DTT, 250 mM imidazole). Zuo1 containing fractions were identified by 4%–12% sodium dodecyl sulphate–polyacrylamide gel electrophoresis (SDS–PAGE) and incubated with TEV-protease overnight at 4°C. The cleaved His_6_-tag was removed by immobilized metal ion chromatography using lysis and elution buffer. The protein was further purified by size exclusion chromatography (Superdex 75 column, GE Healthcare) using SEC buffer (300 mM NaCl, 20 mM HEPES, pH 7.0, 1 mM DTT). Analytical SDS–PAGE of all steps are presented in Supplementary Fig. S1. The protein was concentrated using a Vivaspin (molecular weight cut off = MWCO 3 kDa). The protein concentration was determined by a Bradford assay in comparison to known amounts of bovine serum albumin (BSA) as a standard protein. The concentrated Zuo1-containing fractions were buffer exchanged using 25 mM KPi (pH 7.0) or 100 mM NaCl, 10 mM Tris (pH 7.0), and a Vivaspin (MWCO 3 kDa) according to the experimental setting.

### *In vitro* folding and analysis of G4 structures and annealing of control DNA structures

Oligonucleotides were purchased from Eurofins MWG Operon (Ebersberg, Germany) in high performance liquid chromatography (HPLC) grade. After desalting via ultracentrifuge filtration devices (Vivaspin 2, 3 kDa cut-off), oligomers were dissolved in ddH_2_O and stored at −20°C. For the NMR and CD interaction studies, G4 DNA was prepared in 25 mM potassium phosphate buffer (pH = 7.0) or 100 mM NaCl + 10 mM Tris∙HCl (pH 7.0) and incubated at 95°C for 5 min. The DNA was folded by slow cooling (within 1 h) to room temperature.

### NMR spectroscopy

All NMR spectra were recorded on a 600 MHz NMR spectrometer equipped with a TCI Prodigy ^1^H[^13^C, ^15^N] Z-GRD probe. All experiments were conducted at 25°C in K^+^ buffer (25 mM potassium phosphate, pH 7.0) or Na^+^ buffer (100 mM NaCl, 10 mM Tris∙HCl, pH 7.0) supplemented with 5% D_2_O. The spectra were referenced to 4,4-dimethyl-4-silapentane-1-sulfonic acid (DSS ). Chemical shift perturbations (CSPs) [39] were calculated using


\begin{eqnarray*}
{\mathrm{CSP(}}\Delta {{\mathrm{\delta }}}_{{\mathrm{NH}}}{\mathrm{) = }}\sqrt {{{{\mathrm{(0}}{\mathrm{.102}}\Delta {{\mathrm{\delta }}}_{\mathrm{N}}{\mathrm{)}}}}^{\mathrm{2}}{\mathrm{ + (}}\Delta {{\mathrm{\delta }}}_{\mathrm{H}}{{\mathrm{)}}}^{\mathrm{2}}}
\end{eqnarray*}

All NMR spectra have been analyzed by Bruker Biospin software TopSpin 4.0.9 and Sparky 3.114 [40].

### Microscale thermophoresis

Cy5-labeled oligonucleotides were purchased from Eurofins MWG Operon (Ebersberg, Germany) in HPLC grade. MST measurements were performed at 25°C and in K^+^ buffer (25 or 100 mM potassium phosphate, pH 7.0) or Na^+^ buffer (100 mM NaCl, 10 mM Tris∙HCl, pH 7.0) supplemented with 0.05% Tween-20 and 0.4 mg ml^−1^ BSA to saturate unspecific interactions. A 1:1 dilution series of Zuo1_348–433_ was performed (c = 200 µM–6.1 nM) and labeled DNA was added (final concentration: 20 nM). After centrifugation at 2000 × *g* for 60 min, the reaction mixtures were transferred into Monolith NT.115 standard-treated capillaries (Nanotemper). Measurements were carried out using the Monolith NT.115 (Nanotemper), using 40% excitation power and 40% MST power. MST Data were analyzed with MO.Affinity Analysis (version 2.1.2).

### Single molecule total-internal reflection

smFRET experiments were performed as described previously [41]. All experiments were performed on a prism-type total-internal reflection microscope using an inverted microscope (Olympus IX71). A 532-nm laser (Crystalaser) was used for Cy3 excitation and images were collected on a back-illuminated Ixon EMCCD camera (Andor, 512 × 512 pixels). Cy3 and Cy5 emissions were separated using dichroic mirrors (640DCLP dichroic mirror, Chroma Technology) and imaged onto the left (Cy3) and right (Cy5) half-chip of the EMCCD camera. Images were corrected for optical aberrations and inhomogeneous evanescence wave illumination using custom-build routines in IDL software (Exelis). An IDL-based mapping algorithm was used to correlate the position of Cy3 and Cy5 signals from the same immobilized molecule, and to extract the time-dependent trajectory for each dye and for each molecule.

### smFRET

The Cy3-labeled DNA strand containing the G4 structure, and the complementary Cy5-Biotin labeled DNA strand were hybridized (G4_IX_ DNA) in 25 mM K^+^ or 100 mM Na^+^ and diluted in 50 mM Tris and different concentrations of NaCl or KCl; 10–50 pM of G4_IX_ DNA was immobilized through the biotin moiety to a slide passivated with streptavidin. The imaging buffer contained 50 mM Tris, 1 mM Trolox, 6.25 mM 3,4-protocatechuic acid and 250 nM protocatechuate dioxygenase and varying concentrations of NaCl and KCl. Tested concentrations of Zuo1 were prepared in the imaging buffer with the appropriate concentration of K^+^ and injected into the channel containing previously immobilized G4_IX_ DNA. Apparent FRET efficiencies after background correction were calculated using (I_A_/[I_A _+ I_D_]), where I_A_ is the acceptor emission intensity and I_D_ is the donor emission intensity. smFRET population histograms were calculated by averaging the FRET efficiency for the first 15 frames from each trajectory. Single-molecule dwell times were extracted using a custom-built Matlab routine as previously described [41]. All measurements were carried out at room temperature (20°C) with frame integration times of 100 or 300 ms depending on sample dynamics.

### Slide passivation

Slides were passivated as described previously [42]. Aminosilane-treated slides and coverslips were passivated for 1–3 h with a mixture of biotin-PEG-SVA and PEG-SVA (Laysan Biosciences) in a mass ratio of 1:100 respectively and dissolved in 100 mM sodium bicarbonate (pH 8.5). A second passivation round was performed for 30 min using 25 mM MS4-PEG (Thermo Fisher Scientific) diluted in bicarbonate buffer (pH 8.0) to limit protein–surface interactions. Slides and coverslips were rinsed well with methanol and water and were subsequently assembled into four channels. Channels were coated with 0.2 mg ml^−1^ streptavidin for 10 min prior to addition of biotinylated FRET labeled DNA.

### Restriction free cloning

The pBG1805 + Zuo1-OE plasmid (Yeast ORF library, Horizon Discovery) containing a GAL1-inducible promotor with URA as selective yeast marker as well as an HA-tag was chosen for these experiments. To perform restriction-free cloning, primers were designed for either deleting the entire Zuo1 protein or retaining only the C-terminal region. These primers were intended for use with the BG1805 + Zuo1-OE plasmid. Polymerase chain reaction (PCR) was carried out with Phusion Flash polymerase (Thermo Fisher) and the PCR product was subsequently digested with *DpnI* (NEB). The newly created plasmids were transformed into DH5α cells, isolated and plasmid sequence was confirmed by sequencing (Eurofins genomics). Plasmids were transformed into wildtype (WT) and *zuo1Δ* cells using the LiAc/SS Carrier DNA/PEG method [43] and cells were plated on SC-URA media.

### Western blot

The strains used for growth assays are listed in Supplementary Table S1. Strains were grown O/N in selective media SC-URA + raffinose (2%) and inoculated the next day in SC-URA + galactose (2%) and grown at 30°C, 200 rpm until OD_600_ = 0.5 was reached. Protein isolation was carried out using the TCA method. Proteins were separated by sodium dodecyl sulphate (SDS) page and blotted on a nitrocellulose membrane. The membrane was stained with antibodies against HA-tag (Santa Cruz #sc-7392) and histone 3 (abcam #ab1791). Fluorescent secondary antibodies were used for detection in the ChemiDoc system (Bio-Rad).

### Spot assay

The strains used for growth assays are listed in Supplementary Table S1. Strains were grown O/N in selective media SC-URA + Raffinose (2%). The next day cells were grown in SC-URA + Galactose (2%) at 30°C, 200 rpm until OD_600_ = 0.5 was reached. 200 µl were transferred to a sterile 96-well plate. Five-fold serially diluted samples were plated as 5 µl droplets on SC-URA + Galactose (2%) plates and UV treatment (15 mJ cm^-2^, 254 nm) was carried out. Cells were grown for 2–3 days at 30°C to evaluate the viability.

### ChIP-quantitative PCR analysis

ChIP experiments were performed as described previously [44]. Briefly, asynchronous cultures were grown to OD_600_ = 0.6 and crosslinked with 1% formaldehyde for 10 min followed by quenching the crosslinking by the addition of 125 mM glycine. Cellular pellet was washed before with ice cold HBS buffer (50 mM HEPES, pH 7.5, 140 mM NaCl) and then with ChIP lysis buffer [50 mM HEPES, pH 7.5, 140 mM NaCl, 1 mM ethylenediaminetetraacetic acid (EDTA), pH 8.0, 1% IGEPAL, 0.1% sodium deoxycholate]. Pellet was resuspended with ChIP lysis buffer with 1 x PIC (Sigma #P8340), snap-chilled in liquid nitrogen and stored at −80°C. Frozen cell pellets were thawed, and cells were lysed using glass beads in Fastprep (MP Biomedicals) in three rounds (60 s, 6.0 m s^−1^). Chromatin was sheared to 200–1000 bp using a Bioruptor (Diagenode) with these settings: high intensity, 30 s ON, 30 s OFF, seven cycles. Shearing quality was estimated on an 1% agarose gel; 400 ng Myc-antibody (Cell signaling #2276) or 800 ng HA-antibody (Santa Cruz #sc-7392) was added to the sheared chromatin and incubated at room temperature (RT) for 1 h followed by incubation with 50 µl Dynabeads-Protein G (Thermo Scientific #10009D) for 1 h. Beads were washed sequentially with SDS buffer, high-salt buffer, Tris–Lithium buffer and Tris–EDTA buffer to remove nonspecific bound DNA. Immunoprecipitated DNA was eluted using Tris–EDTA + 1% SDS followed by incubation at 65°C to reverse the crosslink. Immunoprecipitated DNA was purified using a PCR purification kit (Qiagen ref #28106) and used for subsequent analyses. Quantitative PCR (qPCR) was performed using SyBr Green (Bio-Rad) and fold enrichment of binding regions was quantified using the IP/Input method. Primers used for qPCR are listed in Supplementary Table S3. GraphPad Prism was used to plot the graphs and *P*-value was calculated using one-way Analysis of Variance (ANOVA). Asterisks indicate statistical significance: *P <.05, **P <.01, ***P <.001, ****P <.0001. Plotted results were based on the average of *n* = 3 biologically independent experiments. qPCR were performed according to MIQE guidelines.

## Results

### Zuo1 C-terminus resembles the structured regions of full length Zuo1

**Figure 1.**
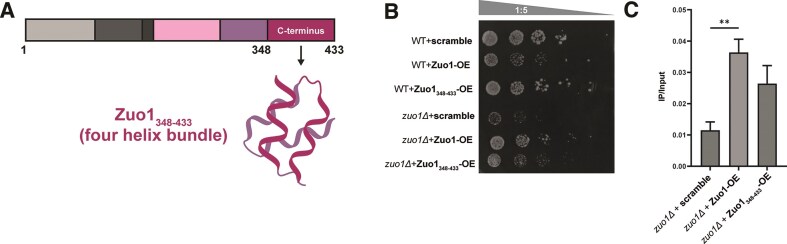
(**A**) Schematic representation of the C-terminal region of Zuo1 (Zuo1_348–433_), which is located between the amino acids 348 and 433. The C-terminus is forming a four helix bundle (pdb: 2LWX) [35] (**B**) Serial dilution of WT and *zuo1Δ* yeast cells containing either an empty plasmid (scramble), Zuo1-overexpression (OE) plasmid or Zuo1_348–433_-OE plasmid spotted on a selective medium. The cells were treated with 15 mJ cm^-2^ UV (254 nm). (**C**) Zuo1-ChIP followed by qPCR. Assays were performed in *zuo1Δ* cells containing plasmids expressing only an HA-tag (scramble), Zuo1-OE-HA, or Zuo1_348–433_-OE-HA. The binding to the G4_IX_ region within the yeast genome was checked. Plotted are the means immunoprecipitate (IP) over input DNA in *n* = 3 biologically independent experiments. Error bars present ± standard error of the mean (SEM). Significance was calculated based on ordinary one-way ANOVA. Asterisks indicate statistical significance: *P <.05, **P <.01, ***P <.001.

We heterologously expressed and purified full-length ^15^N-labeled Zuo1 and monitored its folding by heteronuclear ^1^H, ^15^N NMR correlation experiments (BEST-TROSY) [45]. The 2D spectra featured well-resolved backbone amide resonances together with NMR signals indicative of intrinsically disordered regions in the full-length protein (Supplementary Fig. S2A). The C-terminus of Zuo1 is positively charged, adopts a 4-helix bundle structure (pdb: 2LWX), and is crucial for activating the transcription factor Pdr1 [35]. We heterologously expressed and purified the C-terminus of Zuo1 (Zuo1_348–433_, Fig. 1A). Both NMR spectra of Zuo1 full-length (Zuo1_FL_) and Zuo1_348–433_ showed that the C-terminus is well folded as evidenced by high NMR signal dispersion (Supplementary Fig. S2B). The NMR signals of the C-terminus overlapped with those of Zuo1_FL_ and showed a similar folding. CD spectroscopy supported NMR results and revealed a high α-helical structure content for both, Zuo1_FL_ and Zuo1_348–433_ (Supplementary Fig. S2).

### Zuo1 C-terminus binds to G4_IX_ and affects its conformation

In yeast, Zuo1 binds to G4s [33]. In the absence of Zuo1, less G4 structures can be detected both by immunoblotting and chromatin immunoprecipitation (ChIP). Zuo1-deficient cells are sensitive to UV light and show defects in NER. The defects, observed in response to Zuo1 deletion can be partially rescued by stabilizing G4s through the addition of the G4-binding ligand PhenDC_3_, indicating that G4 destabilization is responsible for these defects [33]. To test whether these Zuo1-dependent effects are primarily resulting from the binding of its C-terminus domain to G4 structures in cells, we expressed Zuo1_348–433_ in wild type yeast cells (WT) and Zuo1-deficient cells (*zuo1Δ*) (Supplementary Fig. S3). Zuo1 binding to G4s after UV treatment supports cell growth and DNA repair [33]. Consequently, *zuo1Δ* cells show a growth defect after UV treatment [33]. Building on this evidence, we hypothesized that if Zuo1_348–433_ binds to G4s and performs functions similar to those of Zuo1_FL_, it should rescue the defects caused by *zuo1Δ*. We monitored cell growth after UV treatment by spot assay in WT cells, in *zuo1Δ* cells, and in cells that expressed Zuo1_348–433_. A serial dilution (1:5) of yeast cells was spotted on rich medium (Fig. 1B). Changes in growth were detected by colony formation. After UV treatment, we observed a more severe growth defect in *zuo1Δ* than in WT cells. This defect was rescued by Zuo1_FL_ and partly rescued by Zuo1_348–433_ expression (Fig. 1B). This observation suggests that the C-terminus of Zuo1 can fulfill partially the function of Zuo1_FL_ in cells. To further test whether Zuo1_348–433_ also binds to G4 *in vivo*, we conducted ChIP followed by qPCR analysis. Zuo1_FL_ and Zuo1_348–433_ were endogenously tagged with HA, and their binding to endogenous G4_IX_ was assessed by ChIP and qPCR. As a control, to determine nonspecific binding, we used cells containing a plasmid (scramble) expressing only the HA-tag (Fig. 1C). ChIP followed by qPCR revealed that both Zuo1_FL_ and Zuo1_348–433_ bind significantly to G4_IX_. Note, the binding of Zuo1_348–433_ to G4_IX_ is lower than that of Zuo1_FL_ (1.3×), but it is still 2.3-times higher than the control (scramble). The obtained results demonstrated that the Zuo1 C-terminus can bind to G4_IX_  *in vivo* and fulfills similar cellular functions as Zuo1_FL_ in cells. Based on this finding, we performed all subsequent experiments with Zuo1_348–433_.

**Figure 2.**
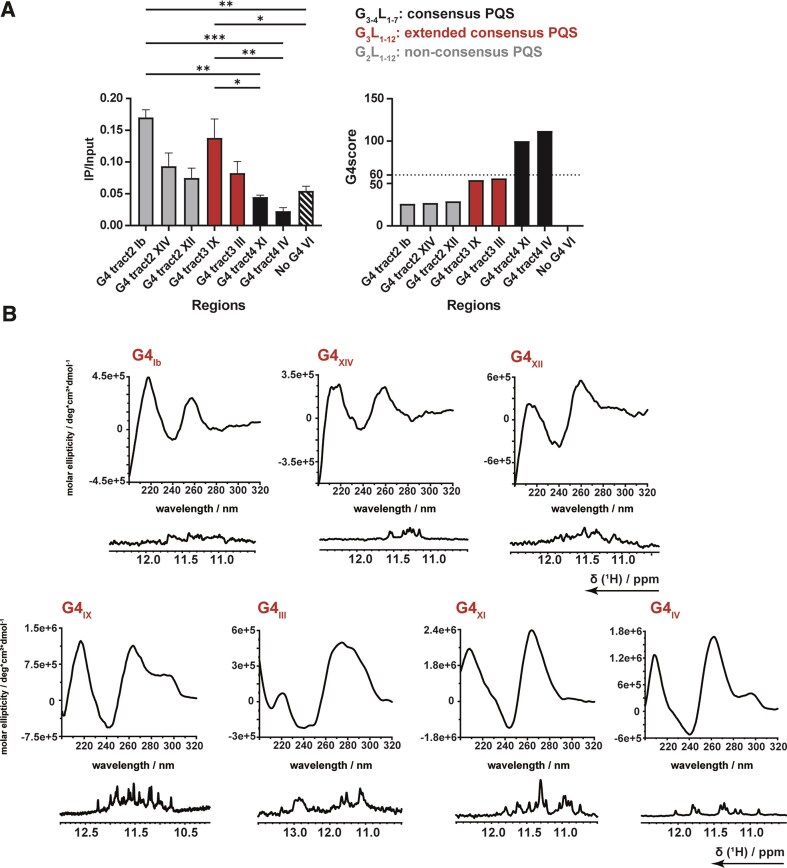
(**A**) ChIP analysis followed by qPCR, showing the binding of Zuo1 to G4 regions with different G-tract numbers and No-G4 control (No G4 VI) (left). qPCR primers are listed in Supplementary Table S3. Plotted are the mean IP over input values in *n* = 3 biologically independent experiments. Error bars present ± SEM. Significance was calculated based on ordinary one-way ANOVA. Asterisks indicate statistical significance: *P <.05, **P <.01, ***P <.001. Right, the G4 scores of each potential quadruplex-forming sequence (PQS) is depicted as calculated by pqs-finder. Light gray PQS are defined as nonconsensus PQS, red as extended consensus PQS, and black as consensus PQS, whereas No-G4 control is striped. A G4 score of 60 (dotted line) was set as the threshold for Zuo1 to bind successfully to these PQS. (**B**) CD spectra (top) and 1D ^1^H-NMR spectra (bottom) of the sequences G4_Ib_, G4_XIV_, G4_XII_, G4_IX_, G4_III_, G4_XI_, and G4_IV_ in the presence of 25 mM KPi-buffer recorded at 25°C. Used sequences are depicted in Supplementary Table S2.

In yeast, mainly nonconsensus G4 motifs exist. We therefore next tested whether Zuo1 binds with similar efficiency to both consensus and nonconsensus G4s in cells. We divided the nonconsensus G4s into strict nonconsensus G4 with only two guanines in each G-tract (tract2), and extended consensus G4s (extG4), which contain 3 guanines per G-tract with long loops (*n* > 7). Similar to our previous reported approach [33], ChIP followed by qPCR was performed to determine Zuo1 binding to genomic regions. In the present study, we selected seven distinct endogenous G4s from yeast. G4s included three strict nonconsensus G4s (tract2) located on chromosomes I, XIV, and XII (G4_Ib_, G4_XIV_, and G4_XII_). In addition, we selected two extG4s (G4_IX_ and G4_III_; tract3) and two consensus G4 motifs (G4_XI_ and G4_IV_; tract4). The likelihood of folding into a G4 was *in silico* predicted by pqs-finder [46]. As control, a region with no predicted G4 motifs (No-G4_IV_, pqs-score: 0) was chosen (Fig. 2A). ChIP and qPCR analysis revealed that both Zuo1_FL_ and Zuo1_348–433_ binds to nonconsensus G4s and extended consensus 2-8 fold more efficient than to consensus G4s (Fig. 2A and Supplementary Fig. S3). Note, although Zuo1_FL_ and Zuo1_348–433_ showed differences in their binding to selected endogenous G4s, both Zuo1_FL_ and Zuo1_348–433_ have the same tendency to bind stronger to nonconsensus G4s. We determined that Zuo1 bound more efficiently to G4s with a pqs-score of 56 or lower, while those with a pqs-score of 100 showed reduced binding (Fig. 2A, left panel shows Zuo1 binding in yeast, right panel shows predicted stabilities).

To further investigate Zuo1 binding in cells, biophysical characterizations of the seven different G4s were conducted using NMR and CD spectroscopy to analyze their formation and topologies (Fig. 2B). CD can characterize G4s topologies based on their distinctive chiroptical signatures. Parallel structures exhibit one maximum at 260 nm and a minimum at 240 nm. In contrast, antiparallel structures display predominant positive bands at 290 and 240 nm and a negative signal at 260 nm. Hybrid structures are detectable by two positive bands at 260 and 290 nm. In the CD spectra recorded here, the selected nonconsensus G4s (G4_Ib_, G4_XII_, and G4_XIV_) showed maxima peaks at around 260 and 220 nm, indicating adoption of a parallel conformation, but with low amplitudes (Fig. 2B, upper panels). For a G4 with three G4-tetrads, 12 different signals of similar intensity can typically be resolved by NMR. The specific 1D ^1^H-NMR spectra of G4_Ib_ and G4_XII_ revealed imino resonances ranging from 11.0 to 12.0 ppm with strong line broadening. This NMR signal pattern suggests Hoogsteen base pairing, but absence of a distinct, single folded structure. G4_XIV_ showed distinct imino resonances, as well as a low amplitude CD signal. These spectroscopic fingerprints could be due to disordered loops or flanking regions weakening the global CD signal. CD analysis for G4_III_ and G4_IX_ (both extended consensus) exhibited different spectral features indicative of different topological organization. G4_III_ displayed a distinctive and broad CD signal at 275 nm and NMR signals in the Hoogsteen and Watson-Crick region that indicate the presence of triplex-like structures (Fig. 2B, lower panels). G4_IX_ had a characteristic maxima and minima peak distribution that can either be interpreted as a hybrid G4 or a mixture of parallel and antiparallel topology. In the 1D ^1^H-NMR spectrum, more than 12 distinct imino signals were observed. This finding indicates the occurrence of more than one topology stabilized by three tetrads within G4_IX_. The two selected consensus G4s (G4_IV_ and G4_XI_) also showed imino signals in the Hoogsteen base pair region and a CD signal at 260 nm, which strongly indicates a parallel G4 structure (Fig. 2B, lower panels). Taken together, we demonstrated that Zuo1_348–433_ has the ability to bind G4s *in vivo*, preferentially nonconsensus and extended consensus G4s. Biophysical investigations further revealed a broad structural diversity among consensus, extended consensus, and nonconsensus sequences.

### G4 formation supported by Zuo1_348–433_ is salt-dependent

**Figure 3.**
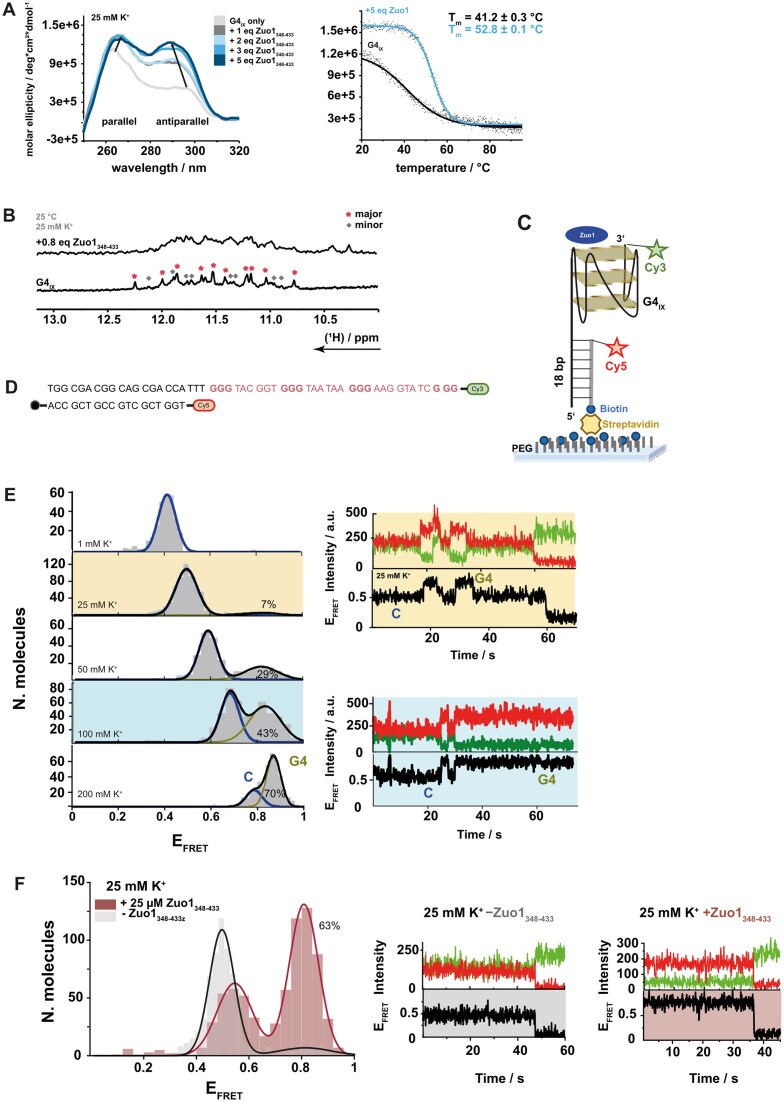
Biophysical characterization of the interaction of the guanosine quadruplex G4_IX_ with Zuo1_348–433_ in the presence of 25 mM K^+^ at 25°C (**A**) CD titration of G4_IX_ with Zuo1_348–433_ at a starting DNA concentration of [DNA] = 7.5 µM. Addition of Zuo1_348–433_ led to a stabilization presumably of both, parallel and antiparallel G4 structures as apparent from CD signal increase at 264 nm and 295 nm, respectively. CD melting curves of G4_IX_ only (black line) and G4_IX_ Zuo1_348–433_ complex (5:1, blue line) showed a stabilization effect of Zuo1_348–433_ on G4_IX_. (**B**) 1D ^1^H-NMR spectra of G4_IX_ imino ^1^H region at [K^+^] = 25 mM and DNA concentration of 100 µM. In the absence of Zuo1_348–433_, the number of imino signals indicated the coexistence of two conformations of G4_IX_ as indicated by * and ♦ in slow exchange. Addition of 0.8 eq Zuo1_348–433_ led to broadening of the NMR signals, in line with a shift in the conformational refolding from slow to intermediate exchange. (**C**) Illustrative diagram of the single-molecule construct used for single-molecule studies depicting the relative positioning of the donor (Cy3, green) and the acceptor (Cy5, red) fluorophores and the biotin moiety used for immobilization to the microscope slide. PEG indicates the polyethylene glycol passivation layer used to prevent nonspecific binding of Zuo1 to the surface. (**D**) Sequence of the used oligonucleotide for FRET experiments. (**E**) Left: smFRET histograms for the G4_IX_ sequence as a function of concentration of K^+^. The solid black line represents the fit to a two-Gaussian population distribution and the blue and yellow solid lines indicate the specific contributions of compacted and G4_IX_ folded conformers. Relative percentual contributions extracted from the area under each Gaussian population are also shown. Right, top: Representative single-molecule intensity trajectory (top panel) and derived FRET trace (bottom panel) obtained at 25 mM K^+^ showing anticorrelated transitions between low- and high-FRET states corresponding to compacted and G4_IX_ folded conformers. Right, bottom: Representative single-molecule intensity trajectory (top panel) and derived FRET trace (bottom panel) obtained at 100 mM K^+^ showing anticorrelated transitions between low- and high-FRET states corresponding to compacted and G4_IX_ folded conformers. (**F**) smFRET histograms illustrating Gaussian distributions of E_FRET_ values for molecules in absence (gray) and presence (red) of Zuo1_348–433_ in 25 mM K^+^. The solid lines represent the fit to a two-gaussian population. Representative single-molecule intensity trajectories (green-FRET donor; red-FRET acceptor) and corresponding smFRET trace (black) obtained at the conditions indicated above. The events at 48 and 39 s were due to Cy5 photobleaching resulting in the concomitant increase in Cy3 signal. The single-step photobleaching and anticorrelated signals of Cy3 and Cy5 were taken as evidence of trajectories arising from single immobilized G4s.

CD titration experiments were performed to test the impact of Zuo1 on all selected G4 structures (Fig. 3A and Supplementary Fig. S4). Among these, only G4_IX_ displayed changes in the CD spectrum at 260 and 295 nm. None of the other selected G4 structures showed evidence of structural reorganization induced by Zuo1_348–433_. For this reason, all subsequent studies were performed with G4_IX_.

G4 topology is known to be affected by different salt concentrations. Sodium and potassium cations (Na^+^, K^+^, respectively) support G4 formation [47]. Protein binding to G4s could exhibit preferential specificity to one topology over others, thus it is important to characterize the influence of the type and concentration of monovalent ions on the structure and dynamics of G4 topologies. To gain insights how Zuo1_348–433_ binding changes the topology of G4_IX_, we first tested the topology of G4_IX_ alone and added K^+^ or Na^+^, respectively. In 25 mM K^+^, G4_IX_ has two topologies: a parallel (260 nm) and an antiparallel (295 nm) state, while in the presence of Na^+^, G4_IX_ has a maximum at 260 nm and is folded into a parallel G4 (Fig. 3A and Supplementary Fig. S5A and B). In the presence of both cations, CD signals indicated the formation of both an antiparallel and parallel population (Supplementary Fig. S5C). Next, we monitored changes in the CD spectra upon titrating Zuo1_348–433_ to G4_IX_ in the presence of K^+^_._ We observed a significant increase in the CD signals of G4_IX_ at 260 nm and 295 nm corresponding to parallel and antiparallel conformations, respectively (Fig. 3A). The relative larger change upon K^+^ addition observed for the 295 nm peak suggests that Zuo1_348–433_ not only recognizes both topologies but also that upon binding Zuo1_348–433_ induces a conformational shift resulting in a higher population of G4_IX_ in the antiparallel state. In the presence of 100 mM Na^+^, Zuo1_348–433_ also binds to G4_IX_ and induces a conformational shift from a parallel to an antiparallel G4 structure (Supplementary Fig. S6A). The binding is also observable in ^1^H, ^15^N BEST-TROSY experiments where the protein residues A349 and N389 experienced maximal CSPs (Supplementary Fig. S6B and C). In the presence of Na^+^ only, G4_IX_ exhibited a broad imino proton region (Supplementary Fig. S6D), indicating conformational heterogeneity. When conducting smFRET in 100 mM Na^+^, we observed a shift toward a lower FRET state, indicating a less compact structure (Supplementary Fig. S6E). We further performed 1D ^1^H NMR-spectroscopy in the presence of 25 mM K^+^. In agreement with CD data, we observed that G4_IX_ adopted two different conformations that interconverted slowly, as evidenced by a doubling of the observable NMR signals (Fig. 3B). The dominant conformation (indicated by asterisks) was favored at elevated temperatures and the minor conformation (indicated by diamonds, Fig. 3B) was not detectable at 38°C (Supplementary Fig. S7). The strong line broadening of the imino ^1^H signals the Zuo1_348–433_:G4_IX_ complex suggested a conformational exchange taking place in the low millisecond time scale. As a result, the NMR signals broadened beyond detection, and the deoxyguanosine nucleotides interacting with the protein could not be individually assigned. An attempt was made to obtain 2D ^1^H, ^1^H-NOESY experiments of G4_IX_ with the aim of enabling nucleotide-specific resonance assignment. However, the signal-to-noise ratio was low, and even at extended measurement times at high magnetic field over several days, the spectral quality remained insufficient. This finding is interpreted as indicative of the highly dynamic nature of G4_IX_, thus designating it as a “metastable” G4. Dynamic structures are more susceptible to rapid exchange with the surrounding solvent water, which leads to weakening of the imino NMR signals, and consequently leads to a reduced number of observable nuclear Overhauser enhancement contacts involving these imino signals. Thermal melting experiments, detected by CD, revealed a melting at 41.2°C in the absence of Zuo1_348–433_ and a melting at 52.8°C after the addition of five equivalents of Zuo1_348–433_ (Fig. 3A, right panel). NMR and CD data indicated that, in the absence of Zuo1_348–433_, G4_IX_ formed a less stable G4 conformation that required K^+^ to efficiently adopt into G4.

In order to determine changes of Zuo1_348–433_ binding to G4_IX_ using different cations, the binding affinities of Zuo1_348–433_ to G4_IX_ at two different cations conditions were measured by MST. MST revealed binding constants in the low micromolar range (K_D _= 4.9 ± 0.8 µM) with 25 mM K^+^ (Supplementary Fig. S8A). The binding constant in 100 mM Na^+^ was K_D _= 85 ± 17 µM, but the signal did not reach saturation (Supplementary Fig. S8B). These data indicate that Zuo1_348–433_ efficiently binds to G4_IX_ sequences and induces different structural states in a salt-dependent manner.

To gain additional insights into the relative populations and structural dynamics of G4_IX_ and how these are modulated by monovalent cations and Zuo1_348–433_ binding, we performed smFRET experiments using a G4_IX_ construct composed of two strands. The first strand contained the G4_IX_ sequence labeled at its 3′ end with a Cy3 fluorophore serving as the FRET donor. This strand was extended at the 5′ end with a handle sequence that hybridized to a complementary 18-nucleotide strand bearing a Cy5 fluorophore at its 5′ end (FRET acceptor) and a 3′ biotin for immobilization on a streptavidin-coated, PEG-passivated quartz slide (Fig. 3C and D). Experiments were conducted in order to assess the influence of ionic strength and cation type on G4_IX_. To this end, the Cy3-labeled G4 sequence and the complementary Cy5-labeled strand carrying a biotin group were hybridized in a background of 25 mM K^+^ by heating at 90°C and slow cooling overnight. The hybridized sample was immobilized at 20 pM concentration in a background of 1 mM K^+^ and progressively exposed to increasing concentrations of K^+^ ions in the range from 1 to 200 mM K^+^. The *in situ* re-folding of the sample was evaluated by calculating the smFRET histograms at each K^+^ concentration (Fig. 3E). At 1 mM K^+^ (Fig. 3E), the smFRET data displayed a single Gaussian peak centered at E_FRET _∼0.41 ± 0.02. A gradual shift in E_FRET_ values was observed with increasing K^+^ concentration, indicating changes in G4_IX_ conformation (Fig. 3E). In addition, the smFRET histograms obtained at K^+^ concentrations above 25 mM displayed a second population centered at E_FRET_ ∼0.85 ± 0.02 whose relative contribution increased with K^+^ and became the predominant feature at high K^+^ (Fig. 3E, left panel). This second population with a high FRET state contributed only 13% when K^+^ was replaced by Na+ (Supplementary Fig. S9). The smFRET traces for K^+^ concentrations above 25 mM showed clear transitions between the two FRET populations and the dwell-time of the high-FRET state increased with the increase in the concentration of K^+^ ions (Fig. 3E, right panel). We assigned the FRET-shifting population to unstructured G4_IX_ conformations that progressively compact as the ionic strength increases and the high-FRET population as resulting from the formation of K^+^-induced G4_IX_ topologies. The latter is supported by the very small contribution of this high-FRET state (13%) when replacing K^+^ by Na^+^ ions (Supplementary Fig. S9). Subsequently, a similar smFRET experiment was carried out to investigate the impact of Zuo1_348–433_ to the surface-immobilized G4_IX_ refolded *in situ* by addition of 25 mM K^+^. Based on the binding affinity of Zuo1_348–433_ to G4_IX_ determined by MST in a background of 25 mM K^+^ (K_D _= 4.9 ± 0.8 µM) and to ensure saturating conditions, we added a 5-fold excess of Zuo1_348–433_ (25 µM). We observed a significant decrease in the contribution of the low-FRET (E_FRET_ ∼0.49 ± 0.01) state and a concomitant increase of a high-FRET state (E_FRET _∼0.81 ± 0.03) from 7% to 63% in the presence of Zuo1_348–433_ (Fig. 3F). Interestingly, the high-FRET state induced by Zuo1_348-433_ binding exhibited an identical FRET value to that promoted by the addition of higher concentrations of K^+^ in the absence of Zuo1_348–433_ (Fig. 3E, left panel). We interpreted this finding as evidence that the high-FRET population represents, in both conditions, an identical state. Taken together the evidences provided by CD, NMR, and smFRET, we assigned this high FRET population to most likely represent an antiparallel folding topology of G4_IX_. These data revealed that at 25 mM K^+^ only 7% of G4_IX_ was folded. After the addition of Zuo1_348–433_ 63% folded into G4 structures, indicating that Zuo1_348–433_ stabilizes G4_IX_. Most of the unstructured states are being actively folded through the interaction with Zuo1_348–433_ and this active folding role agrees with the increase in folding rate we later observed when calculating the dwell times.

### Zuo1_348–433_ accelerates the folding rates of G4_IX_

**Figure 4.**
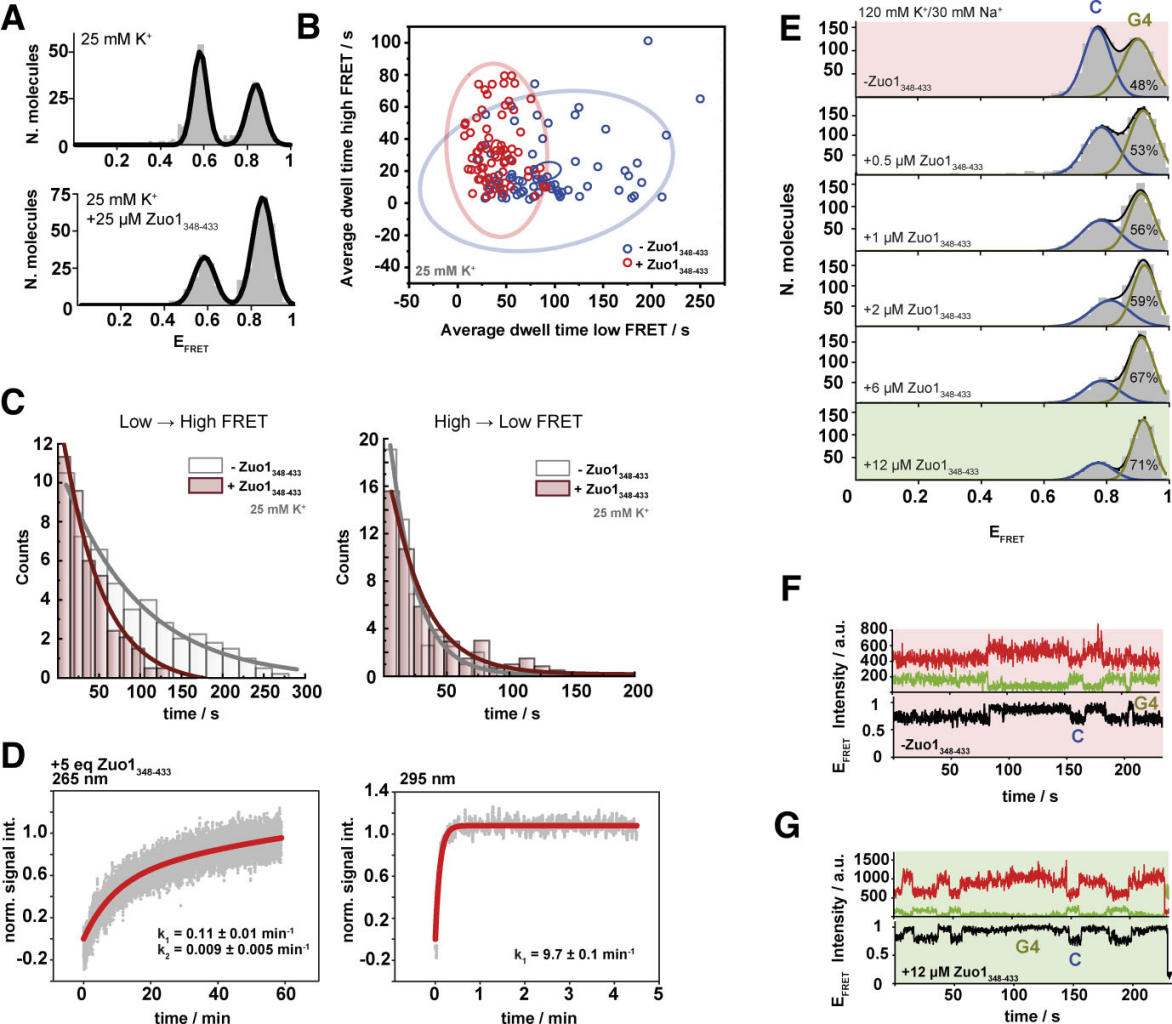
(**A**) smFRET histograms illustrating Gaussian distributions of E_FRET_ values for molecules in absence and presence of Zuo1_348–433_ in 25 mM K^+^. G4-quadruplexes were generated by heating at 90°C for 2 min in a background of 25 mM K^+^ followed by slow cooling to room temperature. smFRET experiments were carried out preserving the 25 mM K^+^ background during the immobilization and data collection steps. The solid lines represent the fit to a two-gaussian population. (**B**) 2D scatter plot showing the average dwell time in seconds of each molecule in high FRET state (y-axis) and in low FRET state (x-axis) with (red) and without (blue) the presence of Zuo1_348–433_. Curves represent the 95% confidence ellipse with (red) and without (blue) Zuo1_348–433_. (**C**) Histograms depicting dwell-times in seconds of molecules transitioning from low to high FRET (left) and from high to low FRET (right), both in the presence (red) and absence (gray) of Zuo1_348–433_. (**D**) Kinetics of the stabilization of the parallel (265 nm) and the antiparallel (295 nm) G4_IX_ signal in the presence of K^+^ after addition of five equivalents Zuo1_348–433_. Kinetic rate constants were taken from the normalized CD signal values. Data have been fitted by bi- or mono-exponential regression. (**E**) smFRET histograms obtained for the G4_IX_ sequence as a function of Zuo1_348–433_ in a background of 120 mM K^+^ and 30 mM Na^+^_._ The solid black line represents the fit to a two-Gaussian population distribution and the blue and yellow solid lines indicate the specific contributions of compacted and G4_IX_ folded conformers. Relative percentual contributions extracted from the area under each Gaussian population are also shown. (**F**) Representative single-molecule intensity trajectory (top panel) and derived FRET trace (bottom panel) obtained in the absence of Zuo1_348–433_ showing anticorrelated transitions between low- and high-FRET states corresponding to compacted and G4_IX_ folded conformers. (**G**) Representative single-molecule intensity trajectory (top panel) and derived FRET trace (bottom panel) obtained in the presence of 12 µM Zuo1_348–433_ showing anticorrelated transitions between low- and high-FRET states corresponding to compacted and G4_IX_ folded conformers.

We showed that G4_IX_ is stabilized by Zuo1_348–433_ and drives a topology shift towards an antiparallel conformation at low salt concentrations (Figs 1 –3). Next, single-molecule kinetic analysis of the FRET trajectories was performed to determine the dynamic aspects of the interaction between Zuo1 and G4_IX_. Studying the influence of Zuo1_348–433_ on the dynamics of G4_IX_ structures required to establish initially the folding kinetics without Zuo1_348–433_. Given that only 7% G4_IX_ structures folded through the *in situ* re-folding process (Fig. 3E), transitions between unstructured and folded states were very rare. As a result, extracting the interconversion rates between both states was limited by the low number of observed transitions. To increase the number of events observed in the absence of Zuo1_348–433_ and the statistical significant of the extracted rates, we performed the smFRET kinetic experiments using samples hybridized and maintained at 25 mM concentration of K^+^ ions. At these conditions, the sample exhibits a high population of folded structures (41%, Fig. 4A) and a significant increase in the number of transitions observed within a single-molecule trajectory. Note that this procedure provided an initial state before addition of Zuo1_348–433_ that is different from that obtained via *in-situ* refolding where molecules remained mostly unfolded by addition of 25 m K^+^ ions (Fig. 3E and F). Variations in the folding efficiency of G4 structures depending on experimental conditions are well known and have been recently reviewed [48, 49]. Interestingly, independently on the initial state of the sample, the final state obtained upon addition of a similar concentration of Zuo1_348–433_ displays very similar relative populations of fully folded G4 structures. Upon addition of 25 µM Zuo1_348–433_ to ensure binding saturating conditions, we observed a 63% population of structured G4_IX_ through the refolding pathway (Fig. 3F) and 66% in the case samples constantly maintained and immobilized at 25 mM K^+^ (Fig. 4A).

Average dwell times for the high- and low-FRET states obtained in the absence and presence of 25 µM Zuo1_348–433_ are shown in Fig. 4B where each data point corresponds to a single G4_IX_ surface-immobilized molecule. Addition of Zuo1_348–433_ induced a shift towards lower average dwell times of the low-FRET state whilst those for the high-FRET state remained mostly unaltered. These data indicate that binding of Zuo1_348–433_ increased the rate of formation of a more compact G4_IX_ structure but did not affect the stability of this state once it is formed. In addition, we calculated the smFRET histogram of kinetic rates for the low-FRET to high-FRET transition and the reverse (Fig. 4C). In this case, instead of average rates per molecule, the rates for individual transitions were computed independently of the molecule in which they appeared, grouped in a histogram, and fitted to a mono-exponential decay function to extract the corresponding rate. In the absence of Zuo1_348–433_, a value for the rate of transition from the low- to the high-FRET state of 0.011 ± 0.003 s^−1^ was obtained, which increased by ∼2-fold in the presence of 25 µM Zuo1_348–433_ (0.021 ± 0.004 s^−1^). For the reverse process, the rates were very similar with values of 0.050 ± 0.005 s^−1^ with no Zuo1_348–433_ to 0.036 ± 0.006 s^−1^ in the presence of Zuo1_348–433_ (Fig. 4C). This reinforces the interpretation that Zuo1_348–433_ alters the conformational equilibrium of G4_IX_ in K^+^ mostly by accelerating the association to the DNA sequence and promoting its transition to a more compact state.

To directly correlate the impact of Zuo1_348–433_ on the folding kinetics of G4_IX_ with specific topologies, we performed rapid mixing experiments involving the addition of Zuo1_348–433_ to pre-folded G4_IX_ in a background of 25 mM K^+^ ions by CD spectroscopy. To monitor the formation of antiparallel G4s, the CD signal at a wavelength of 295 nm was recorded over time. The mixing revealed that the binding of Zuo1_348–433_ is biphasic at 1–3 equivalents of protein (Supplementary Fig. S10). When adding five equivalents, the second phase is no longer detected and only one rate constant of k = 9.7 ± 0.1 min^−1^ was determined (Fig. 4D). While the noise level of the data at 265 nm is not sufficient to provide an exact rate, the measurements indicate that folding at 265 nm proceeds at a slower rate than at 295 nm, further supporting the idea of conformational landscape for G4_IX_ that, assisted by Zuo1_348–433_, dynamically shifts to populate the antiparallel conformation predominantly. Thus both, K^+^ and Zuo1_348–433_ accelerate folding to the antiparallel topology.

To confirm that the observed ability of Zuo1_348–433_ to induce G4 formation was preserved at more physiologically relevant concentrations of monovalent ions, a smFRET titration at 120 mM K^+^ and 30 mM Na^+^ that mimics the concentration of both ions in human cells was conducted [48]. As shown in Fig. 4E, in the absence Zuo1_348–433_, G4_IX_ populates two conformations with roughly equal stability, a conformation with an E_FRET_ ∼0.78 and a 52% contribution and a high-FRET conformation (E_FRET_ ∼0.92) with a 48% contribution. The higher FRET value is similar to that induced by Zuo1_348–433_ in a background of 25 mM K^+^ (Fig. 3F) and also similar to the predominant structure formed at higher K^+^ ions concentration (200 mM K^+^) (Fig. 3E). Thus, this structure was assigned to the folded G4s and the lower FRET conformation (E_FRET_ ∼0.78) corresponds to a compacted state facilitated by the high ionic strength. Importantly, the addition of Zuo1_348–433_ induced a progressive increase in the folded quadruplex structure from 48% with no Zuo1_348–433_ to 71% at a Zuo1_348–433_ concentration of 12 µM. Representative smFRET traces in the absence and presence of Zuo1_348–433_ are shown in Fig. 4F and G, respectively.

## Discussion

Despite their functional relevance in cells, it is still unknown how the topologies of G4s with different stabilities are linked to biological function. In fact, it is postulated that differences in G4 stability are a functional feature of G4s and exhibit organismal specificity [9, 28, 49]. In yeast, mainly nonconsensus G4 motifs were detected which are, based on computational predictions, presumed to be metastable [24]. The vast amount of nonconsensus G4s both in yeast and also humans raises the question whether also nonconsensus, metastable G4s can modulate cellular processes and whether specific proteins support G4 function by stabilizing specific G4s. Zuo1 is a newly described G4-binding protein in yeast [33]. Here, we characterized its mode of binding to G4_IX_, a known extended consensus G4 from yeast. We determined that the C-terminus of Zuo1 (Zuo1_348–433_) is structured and sufficient for G4 binding both *in vitro* and in cells. Experiments in yeast revealed that Zuo1_348–433_ is sufficient to partially rescue the growth defects of *zuo1∆* cells (Fig. 1B), indicating that the C-terminus can bind G4 motifs and partially substitute the function of Zuo1_FL_ at nuclear G4s. These data were supported by the ChIP experiment (Fig. 1C) showing that the Zuo1 C-terminus binds to G4_IX_. However, Zuo1_FL_ binds stronger than Zuo1_348–433_ (Fig. 1C). Together the yeast experiments indicate that the C-terminus binds to G4s but that the unstructured region at the N-terminus supports G4 binding and function. In addition, we could show Zuo1 binding to seven endogenous G4s in yeast. Note, Zuo1 preferentially binds to nonconsensus and metastable G4s *in vivo* (Fig. 2). Further detailed biochemical and biophysical assays presented here showed that Zuo1_348–433_ stabilizes G4_IX_, induces G4 formation, and drives a topology shift towards the antiparallel conformation (Fig. 3, 4).

Depending on monovalent cation, G4_IX_ forms different topologies: in K^+^, parallel and antiparallel forms coexist, while in Na^+^ only parallel G4s form. Our smFRET analyses revealed a pronounced conformational change upon the addition of Zuo1_348–433_ in the presence of K^+^ (Fig. 3F), with the complex adopting a conformation similar to that observed under elevated K^+^ conditions (Fig. 3E). In contrast, the conformation appeared less compact in the presence of Na^+^ after addition of Zuo1_348–433_ (Supplementary Fig. S6D). These observations suggest that K^+^ promotes a more compact topology, stabilizing a functionally relevant conformation of the complex. This finding is consistent with physiological ionic conditions, where intracellular K^+^ concentrations exceed those of Na^+^. In human cells, K^+^ concentrations typically range from 140 to 150 mM, while Na^+^ concentrations remain between 10 and 15 mM [48]. In yeast, K^+^ concentrations are even higher—∼290–310 mM—due to the absence of a Na^+^/K^+^-ATPase [50, 51], whereas Na^+^ concentrations are around 20 mM [52]. This disparity underscores K^+^ as the predominant monovalent cation within the intracellular environment.

We demonstrated that the interaction between Zuo1_348–433_ and G4 structures, as well as its ability to promote G4 folding, is preserved under these physiologically relevant conditions, with 120 mM K^+^ and 30 mM Na^+^, and occurs with affinities in the low micromolar range (Fig. 4E). Notably, Zuo1_348–433_ exerts differential effects on G4_IX_ depending on the ionic environment, whereas many other G4-binding proteins are selective for specific G4 topologies [53]. This selectivity highlights the unique role of Zuo1_348–433_ not only as a G4-binding factor, but also as a G4 stabilizer that actively shapes the G4-folding landscape. In particular, Zuo1_348–433_ not only stabilizes G4_IX_ but also promotes its formation, as evidenced by both FRET and CD (Fig. 3,4).

In the human genome, consensus G4s with short loops are overrepresented but consist mostly of deoxyadenosine loops, which are the most unstable short loops [54]. This instability, however, can be increasingly recognized as a functional advantage over more stable G4s. While some long-looped G4s, which are considered as more unstable, are folding slower than short-looped sequences [55], the slow unfolding kinetics of certain stable G4s render them less favorable for processes involved in genome maintenance [54]. Accordingly, the polymorphic nature of G4s, rather than a single stable structure, in human promoter sequences is associated with their biological function [56]. Well-studied human G4 forming sequences such as *c-MYC, BCL2, KRAS*, and *VEGF* are more polymorphic than the average of G4 rich sequences [56]. As higher polymorphism also includes metastable states additionally to very stable distinct folds [56], it is possible that regions with less stable nonconsensus G4s contribute to genome stability. The presence of G4-forming sequences in the yeast genome is in contrast rather low. Marsico *et al.* described that the occupancy of G4s with two tetrads and long loops (G_2_L_1–12_) is predominant in yeast, while in the human genome the majority of G4s consist of three G tetrads [23]. Within the last few years, several G4 forming sequences without three consecutive deoxyguanosines within a corresponding G-tract have been reported [12]. These reports initiated discussions of whether these less stable, nonconsensus G4s have the capacity to support or modulate cellular processes such as gene expression or genome stability. The data presented herein support a model in which metastable, nonconsensus G4s can have the capacity to influence or support cellular processes in conjunction with proteins. Due to the vast majority of different G4 motifs and topologies, we anticipate the existence of additional G4 stabilizing proteins that have an impact on G4 function. As an example of this emerging trend of multifaceted proteins interacting with G4 motifs is APE1. APE1 is traditionally linked to base excision repair and regulating gene expression through its redox activity, but has been recently shown to stabilize G4s in cells by direct association, particularly at *KRAS* promoter regions [57] and G4s comprising endogenously oxidized DNA bases [58]. In both scenarios, APE1 was shown to mostly recognize and increase the folding efficiency of parallel G4s. Interestingly, the dissociation constants reported for APE1 are in the low nanomolar range, which is 1000-fold lower than those reported here for Zuo1_348–433_. Thus, although APE1 and Zuo1 share a common G4-stabilizing function, Zuo1 might represent the first example of a promiscuous G4-stabilizing protein capable of interacting and stabilizing a broader range of G4 topologies as revealed in this study; a feature potentially enabled by its moderate affinity towards G4s when compared to APE1.

The high number of G4s with nonconsensus sequences in yeast may indicate a more global cis-acting function of G4s with other genomic and epigenomic features that may require less stable or more dynamic G4 structures for function. In the context of these findings, we speculate that Zuo1 is influencing and regulating the G4 folding equilibrium as well as changes of G4 conformations within the yeast genome by enhancing G4 stability. In this context, it is important to note that we have focused so far on understanding the molecular details of Zuo1 interactions with G4s using monomeric single-stranded G4 structures (NMR, CD) or duplex–G4s (smFRET) without the complementary strand or flanking sequences. Although this approach has dominated biophysical studies of G4s for multiple technical reasons, recent reports [59, 60] have emphasized the importance of incorporating G4s within structures that mimic the genomic layout [i.e. duplex–G4–duplex (DGD)]. For instance, in an elegant study using cryo-EM and SAXS [61], Monsen *et al.* revealed a marked propensity of DGD constructs derived from the *MYC* NHEIII element to form a stacked duplex–G4 interaction at the 5’ G-tetrad interface. However, whether such stacking is specific for parallel structures as those observed in *c-MYC* promoter G4s or if it is preserved across diverse topologies needs further investigation. This is also true for the implications of this arrangement with respect to protein accessibility. More recently, Fleming *et al.* studied the folding of the *VEGF* promoter G4 using a similar DGD arrangement. Interestingly, a drastic decrease (∼200-fold) was observed when comparing the folding kinetics of the *VEGF* DGD (half-life ∼5.3 min) to values reported for single-strand versions and the *VEGF* DGD folding rate was considerably increased upon addition of nanomolar concentrations of APE1 [60]. If a similar decrease of the rates of folding occurs in G4_IX_ DGD structures, this would reinforce the role of Zuo1 described here as a stabilizer and folding enhancer in a cellular context.

In human cells, ZRF1, the orthologue of Zuo1, shares conserved function such as its involvement in the ribosome-associated complex and the regulation of polypeptide folding [62]. Similar to Zuo1, ZRF1 is not confined to the cytoplasm but is also found in the nucleus, where it participates in recruiting NER proteins in particular after UV irradiation [36, 63]. After UV irradiation, ZRF1 binds robustly to G4-forming sequences in the nucleus, which is essential to prevent UV-induced senescence [36]. ZRF1 is overexpressed in various types of leukemia, including acute myeloid leukemia (AML), which correlates with the observed increase in G4 levels in AML cell lines [64]. ZRF1 has not been further characterized as a G4 binding/stabilizing protein, yet. Given the similarities between Zuo1 and ZRF1, it is conceivable that ZRF1 may also play a role in stabilizing and promoting G4 structures. This finding leads to the speculation that OE’s of ZRF1 in cancer may support the elevated G4 levels observed in multiple cancer-related disease [65].

**Figure 5.**
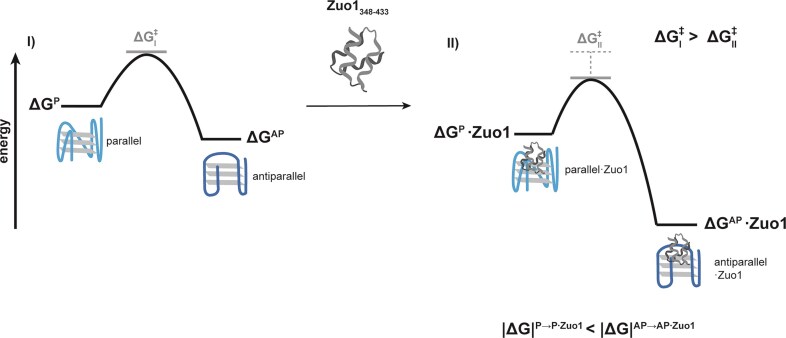
Schematic energy diagrams illustrating the binding of Zuo1_348–433_ to the G4s of G4_IX_. In the presence of K^+^, G4_IX_ exists in a dynamic equilibrium between parallel and antiparallel G4 topologies, which are both stabilized upon binding of Zuo1_348–433_. The interaction with Zuo1_348–433_ facilitates the folding into the antiparallel conformation, resulting in a higher |ΔG| for the antiparallel state relative to the parallel fold. Overall, Zuo1_348–433_ lowers the Gibbs free energy barrier, thereby promoting more efficient access to both conformational states.

In summary, our results demonstrate that Zuo1_348–433_ binds to G4_IX_ and promotes its folding. Unlike many G4-binding proteins, Zuo1_348–433_ has distinct effects depending on the ionic environment, stabilizing both parallel and antiparallel G4_IX_ conformations. Zuo1_348–433_ facilitates the transition into the antiparallel fold (Fig. 5). Our findings support the model in which Zuo1_348–433_ is a distinct G4-binding protein that modulates the folding landscape of G4s in yeast. This opens up the possibility of investigating the role of Zuo1_348-433_ in a cellular context in more depth.

## Supplementary Material

gkaf1055_Supplemental_File

## Data Availability

All cell lines, plasmids can be requested by the authors. See for raw data https://doi.org/10.25716/gude.1x1e-wqs2.
