# Supplementary material for: The Zuo1 C-terminal domain stabilizes DNA guanosine quadruplex (G4) structures located on Chromosome IX in Saccharomyces cerevisiae
Source: Nucleic Acids Res. 2025 Nov 3;53(20):gkaf1055. doi: 10.1093/nar/gkaf1055 (PMC12582019; doi:10.1093/nar/gkaf1055)
Supplement: gkaf1055_Supplemental_File [file gkaf1055_supplemental_file.docx]

**The Zuo1 C-terminal domain stabilizes the DNA guanosine quadruplex (G4) structures located on Chromosome IX in *Saccharomyces cerevisiae***

Ines Burkhart^1,^*, Michaela Limmer^2,3,^*, J. Carlos Penedo^4,5^, Li-Chia Sauer^2,3^, Harald Schwalbe^1#^, Katrin Paeschke^2,3,#^

^1^ Institute for Organic Chemistry and Chemical Biology, Center for Biomolecular Magnetic Resonance, Goethe-University Frankfurt, Max-von-Laue-Str. 7, 60438 Frankfurt/Main

^2^ Department of Oncology, Hematology and Rheumatology, University Hospital Bonn, 53127 Bonn, Germany

^3^ Department of Clinical Chemistry and Clinical Pharmacology, University Hospital Bonn, Bonn, Germany.

^4^ Centre of Biophotonics, Laboratory for Biophysics and Biomolecular Dynamics, School of Physics and Astronomy, University of St Andrews, North Haugh, St Andrews, KY16 9SS

^5^ School of Biology, University of St Andrews, North Haugh, St Andrews, KY16 9ST

**Supporting Information**


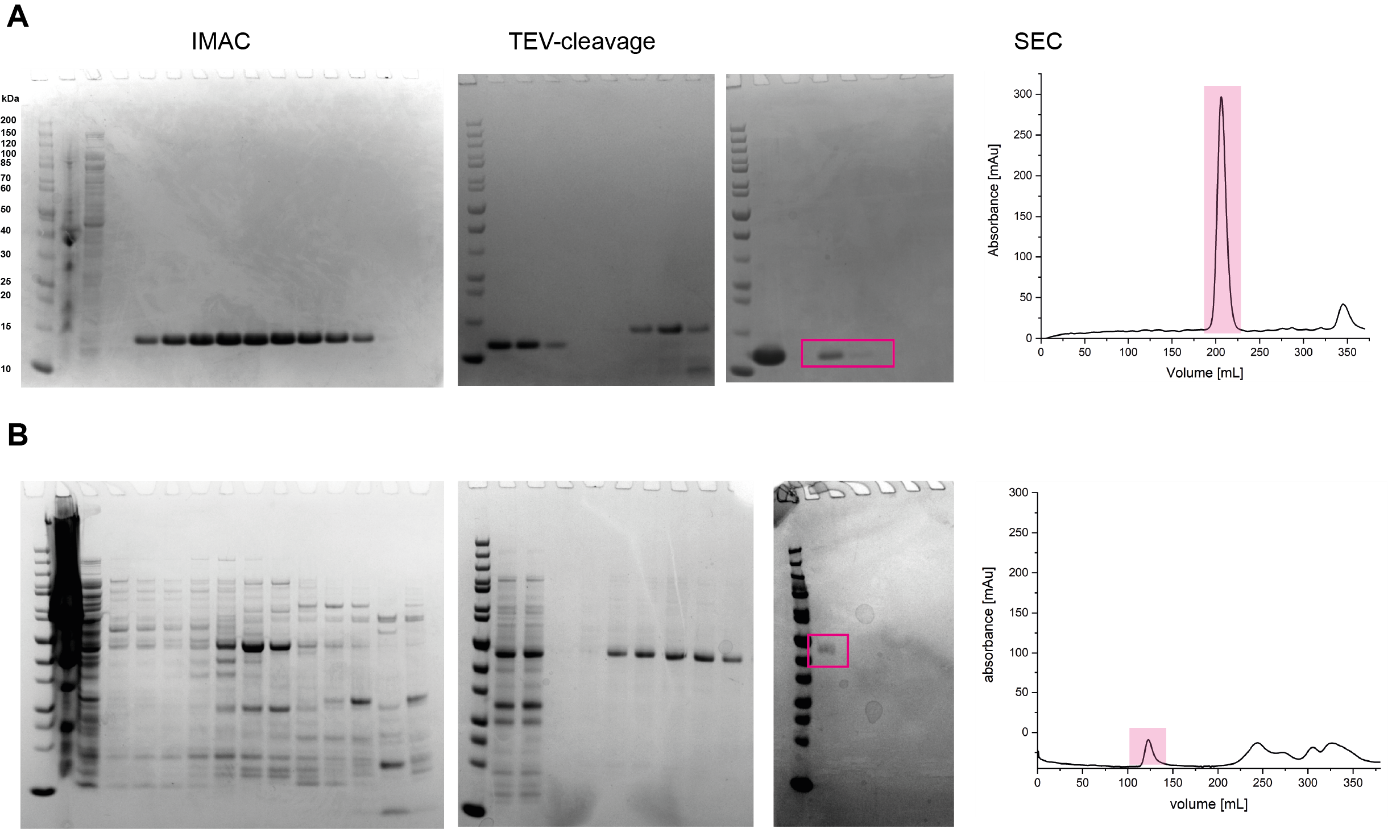


**Supplementary Figure 1 Expression and purification of Zuo1 and Zuo1_348-433_.**

SDS-PAGE of the C-terminal Zuo1_348-433_ (A) and the 433 amino-acid long full-length construct (B). Protein purification included Ni-NTA immobilized metal affinity chromatography (IMAC), cleavage of the TEV-tag and size exclusion chromatography. The proteins of interest are indicated with pink boxes.


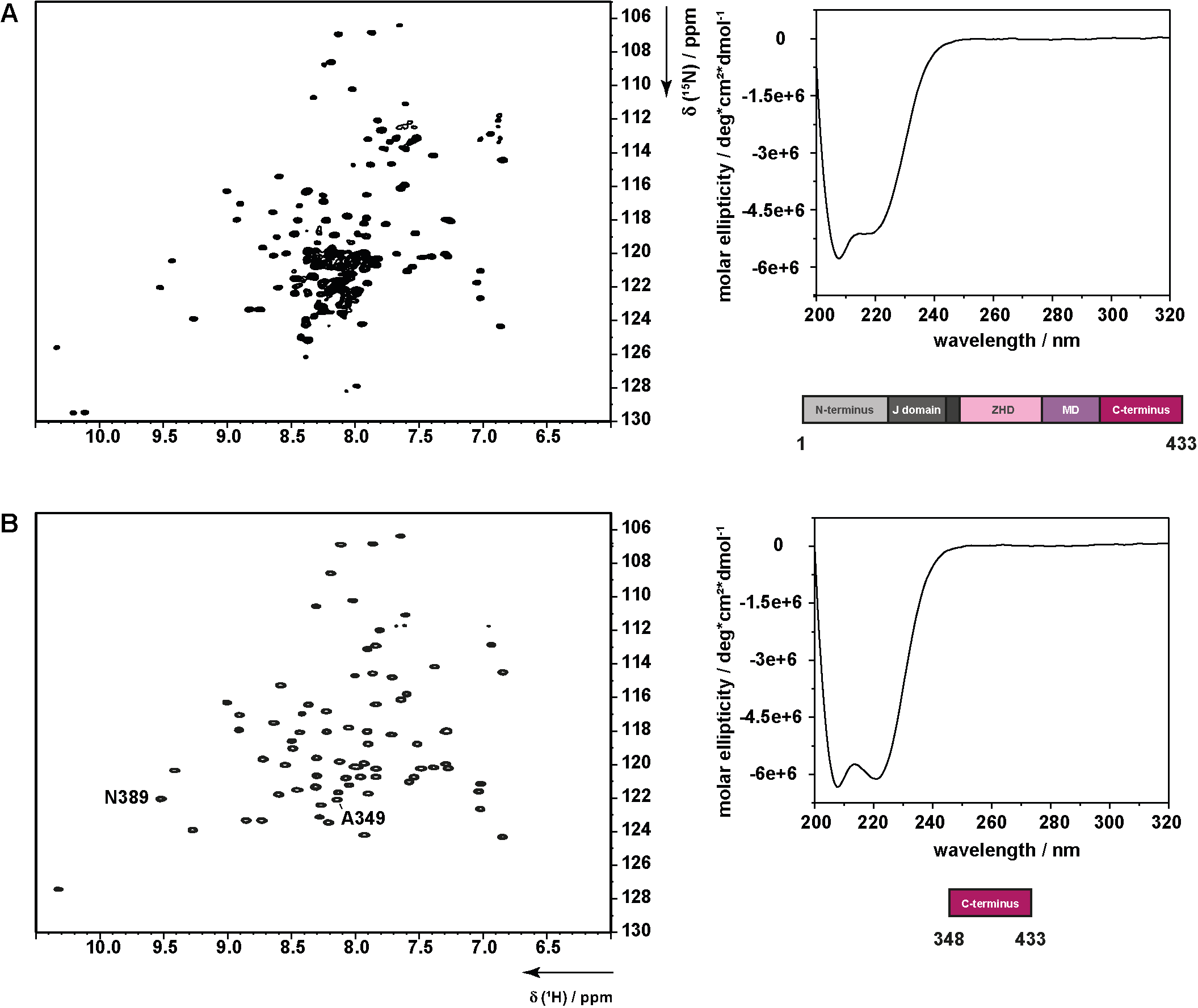


**Supplementary Figure 2 Zuo1 and Zuo1_348-433_ share structural similarities.**

^1^H, ^15^N BEST-TROSY NMR (left), CD (right) spectra and schematic representation of protein domains of A) Zuo1 full length protein and B) Zuo1_348-433_. The NMR signals for the amino acids N389 and A349

have the largest CSP values. All spectra were recorded at 25 °C, NMR spectra were recorded at 600 MHz.


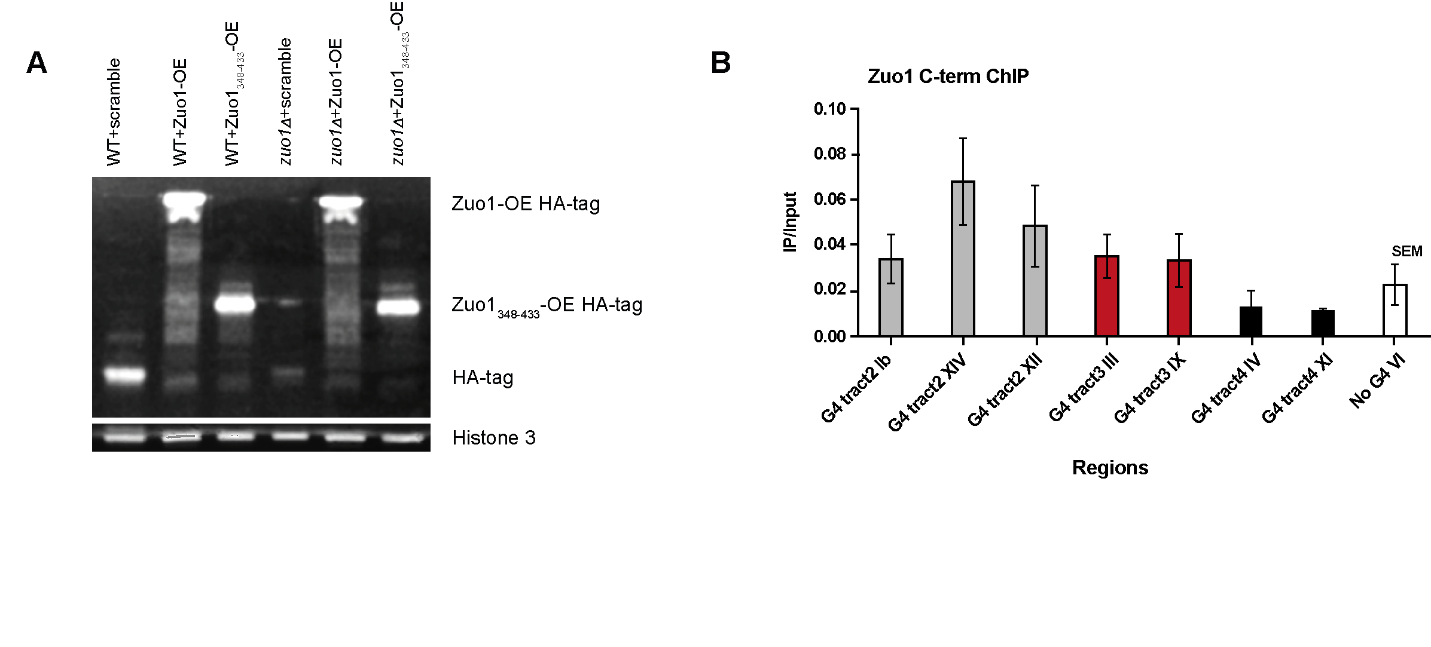


**Supplementary Figure 3 Expression and Binding abilities of Zuo1_348-433_**

A) Exogenously expressed Zuo1 C-terminus rescues growth defect Western blot detecting HA-tags and Histone 3 as housekeeping protein to confirm the expression of Zuo1-OE, Zuo1_348-433_-OE or the sole HA-tag. B) ChIP analysis followed by qPCR, showing the binding of Zuo1_348-433_ to G4 regions with different G-tract numbers and No-G4 control (No G4 VI).


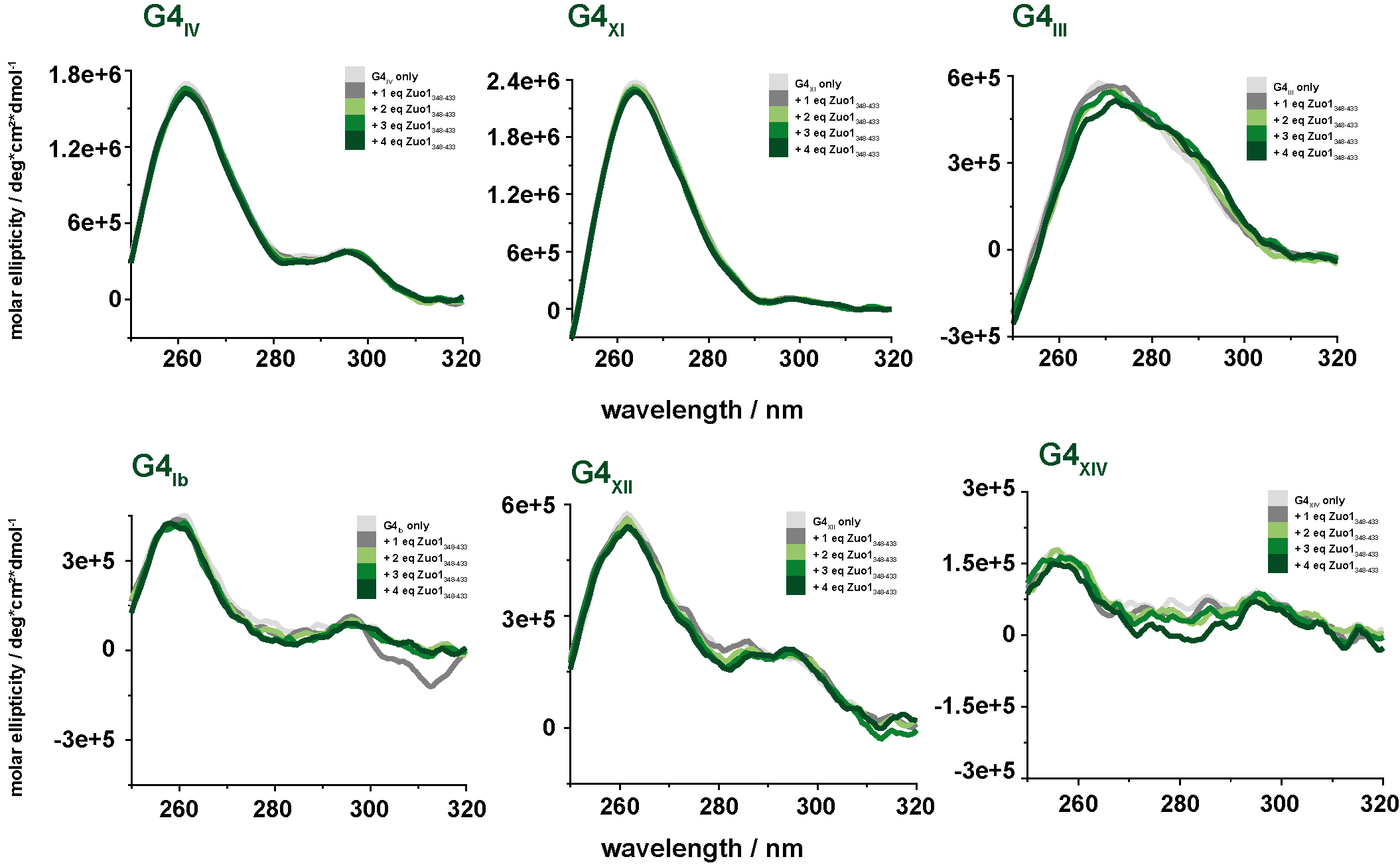


**Supplementary Figure 4 Zuo1_348-433_ does not enhance or stabilize several tested G4 yeast sequences.**

CD titrations of the oligonucleotides G4_IV_, G4_XI_, G4_III_, G4_Ib_, G4_XII_ and G4_XIV_ (7.5 µM) with 1-4 equivalents of Zuo1_348-433_.


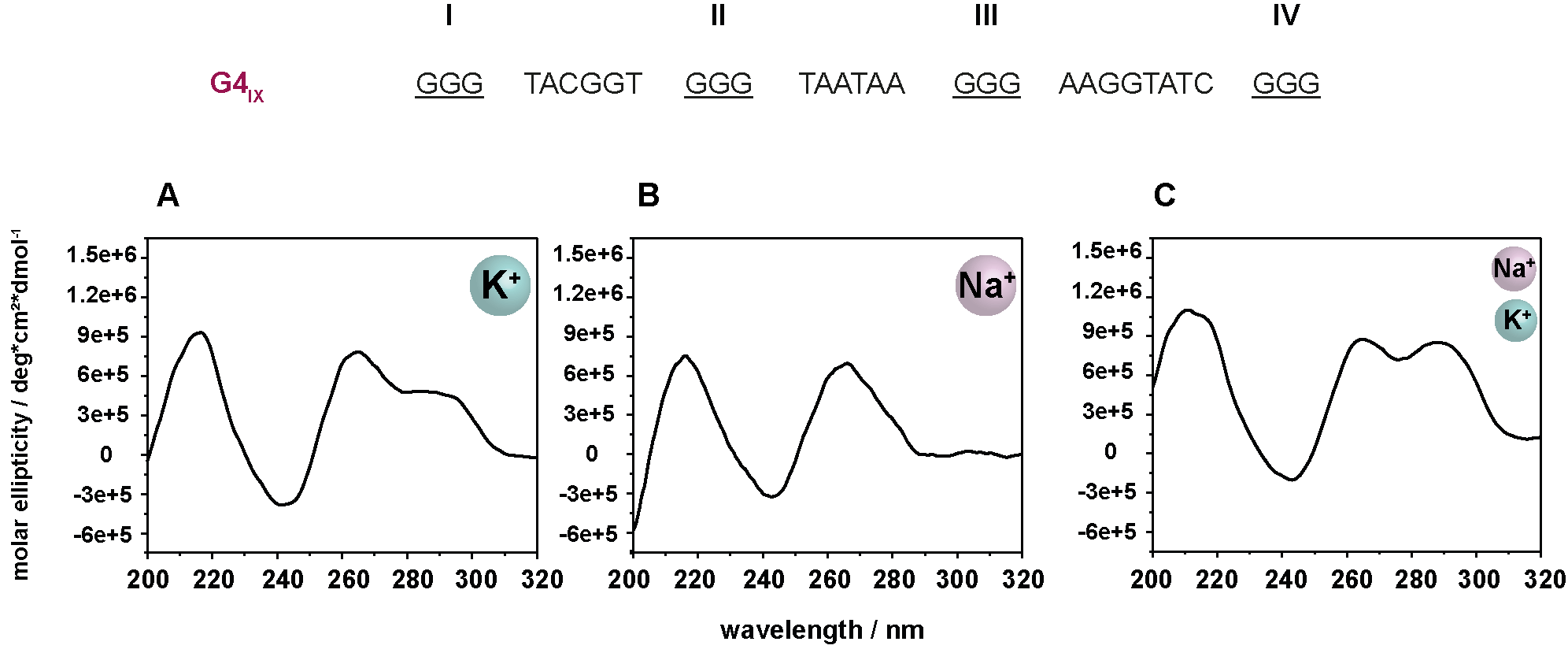


**Supplementary Figure 5 Topology of G4_IX_ is salt dependent (CD).**

CD-spectra of G4_IX_ in the presence of 25 mM KPi-buffer (A), 25 mM NaPi-buffer (B) or both 25 mM Na^+^ and 25 mM K^+^ (C).


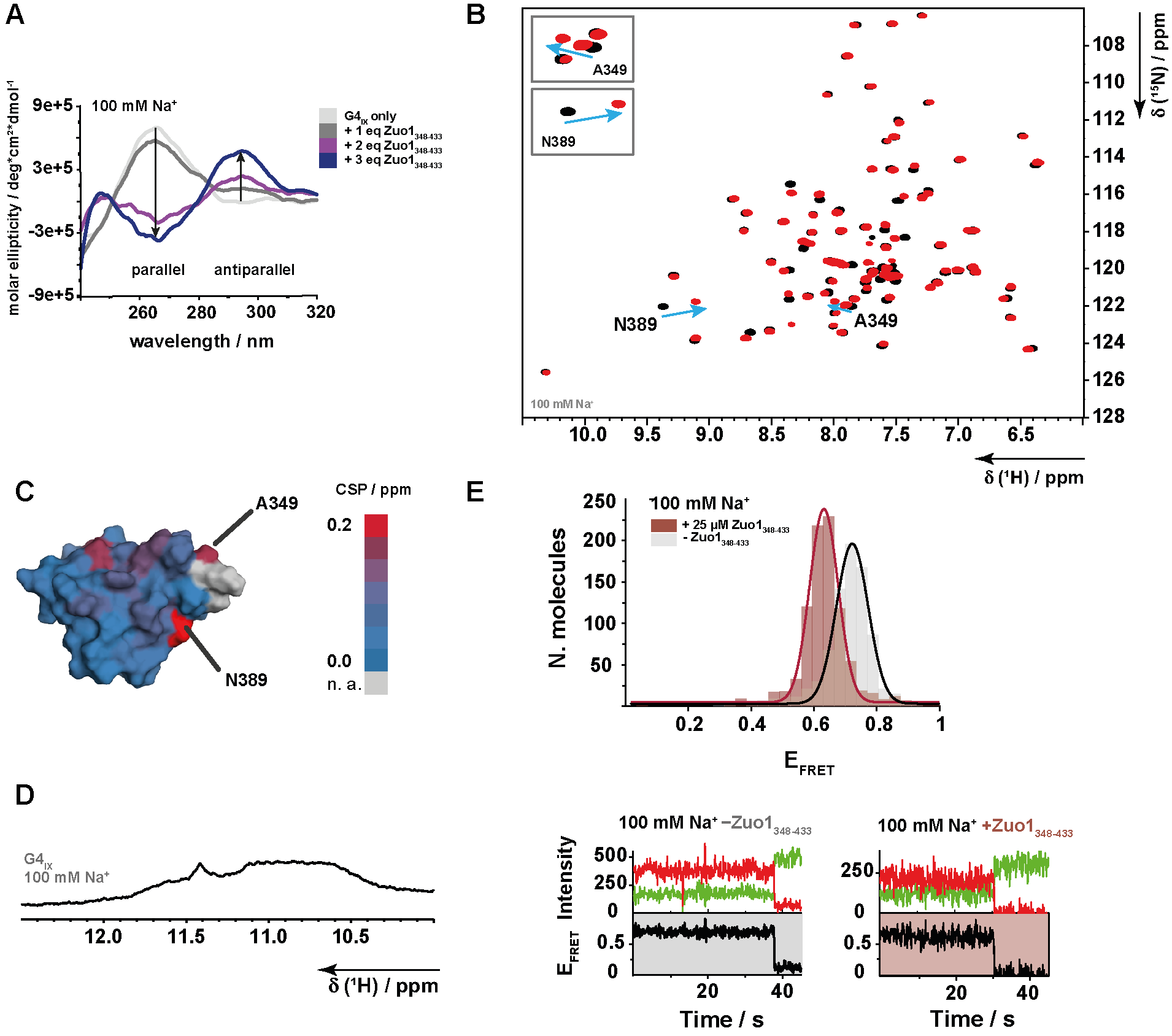


**Supplementary Figure 6 Biophysical investigation of G4_IX_ in the presence of Na^+^ ions**

A) CD titration of G4_IX_ with Zuo1_348-433_ in the presence of 100 mM Na^+^ ions revealed a change of G4 topology from parallel to antiparallel. B) NMR-spectroscopic characterization of the protein-binding site of the C-terminal domain of Zuo1 to DNA quadruplex G4_IX_ (100 µM) at 25 °C. ^1^H, ^15^N heteronuclear correlation experiment (BEST-TROSY) of the ^15^N-labelled C-terminal domain of Zuo1 (Zuo1_348-433)_ alone (black) and in the presence of 0.5 equivalents G4_IX_ (red). C) Chemical shift perturbation (CSP) analysis with colour coding from blue (no CSP) to red (maximal CSP). Chemical shift changes in the presence of 100 mM Na^+^ were mapped on the NMR solution structure of Zuo1 C-terminus (pdb: 2LWX) showing that the terminal amino acids A349 and N389 showed maximal chemical shift perturbations (CSPs) of 0.2 ppm, indicating their involvement in the interaction with Zuo1 (n. a.: not-assigned). The amino acids G346 and S347, which were part of the previous NMR solution structure^[1]^, were not present in the used construct and were therefore not assigned. After addition of G4_IX_, the NMR signal of K348 could not be detected. D) 1D ^1^H-NMR spectrum of G4_IX_ imino ^1^H region at the presence of 100 mM Na^+^. DNA concentration was 100 µM. E) smFRET histograms illustrating Gaussian distributions of E_FRET_ values for molecules in absence (grey) and presence (red) of Zuo1_348-433_ in 100 mM Na^+^. The solid lines represent the fit to a single gaussian population. Representative single-molecule intensity trajectories (green-FRET donor; Red-FRET acceptor) and corresponding smFRET trace (black) obtained at the conditions indicated above. The events at 36 and 43 seconds were due to Cy5 photobleaching resulting in the concomitant increase in Cy3 signal. The single-step photobleaching and anticorrelated signals of Cy3 and Cy5 were taken as evidence of trajectories arising from single immobilized G4s.


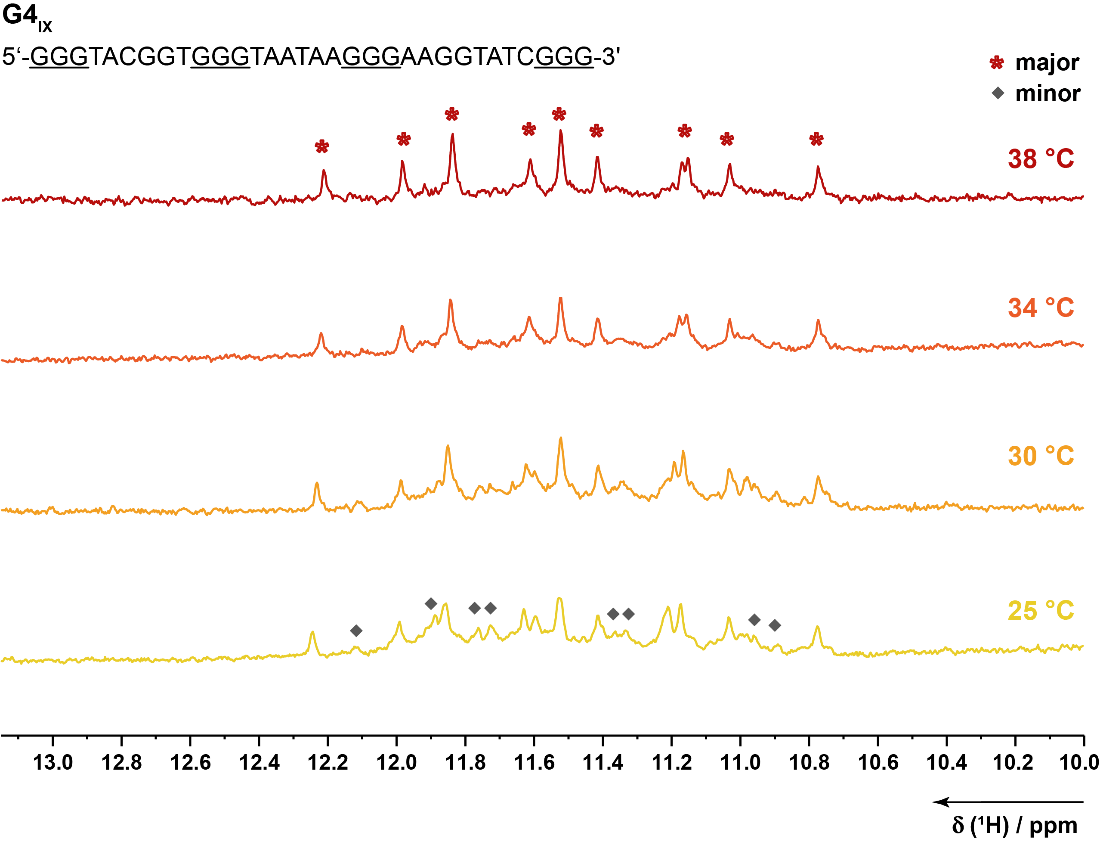


**Supplementary Figure 7 G4_IX_ displays two conformations in NMR.**

1D ^1^H-NMR spectra of G4_IX_ in the presence of 25 mM KPi-buffer recorded at different temperatures from 25 to 38 °C. The two conformations of G4_IX_ are indicated by * and ♦. At 38 °C, the minor conformation was no longer detectable. Spectra were recorded at 600 MHz.


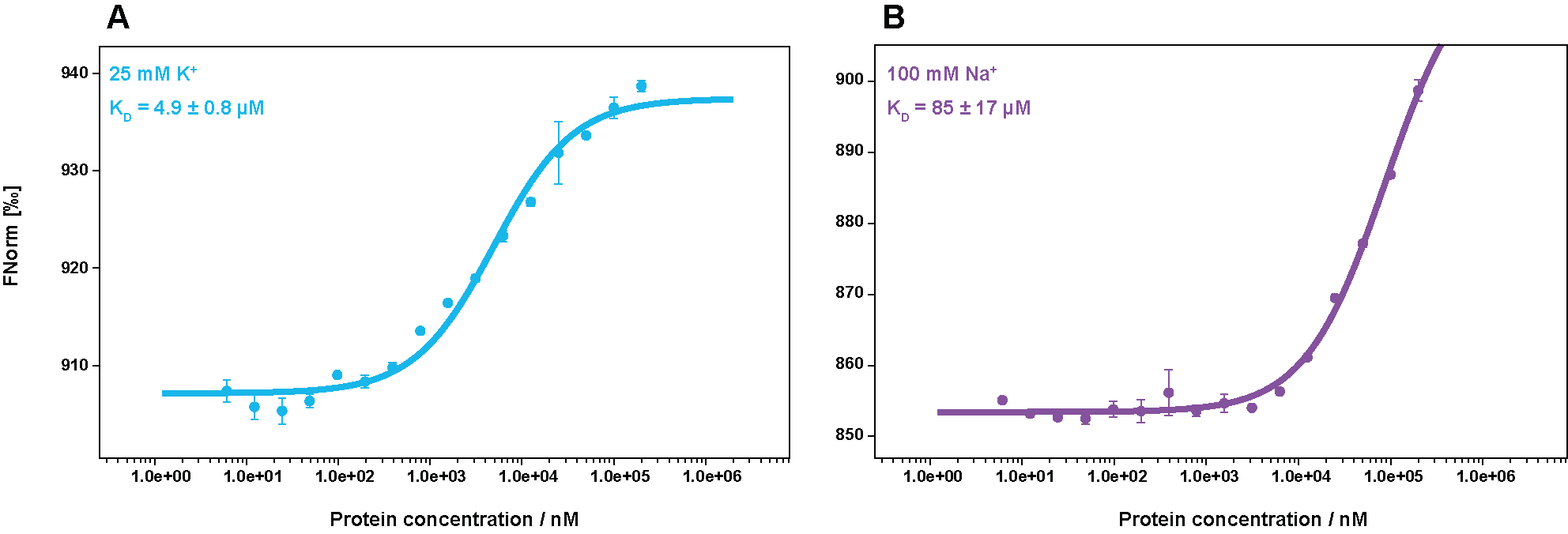


**Supplementary Figure 8 Binding of Zuo1_348-433_ to G4_IX_ occurs in a micromolar range.**

Microscale thermophoresis (MST) of G4_IX_ in the presence of 25 mM K^+^ (A) or 100 mM Na^+^ (B) at 25 °C. K_D_s were determined by sigmoidal fitting.


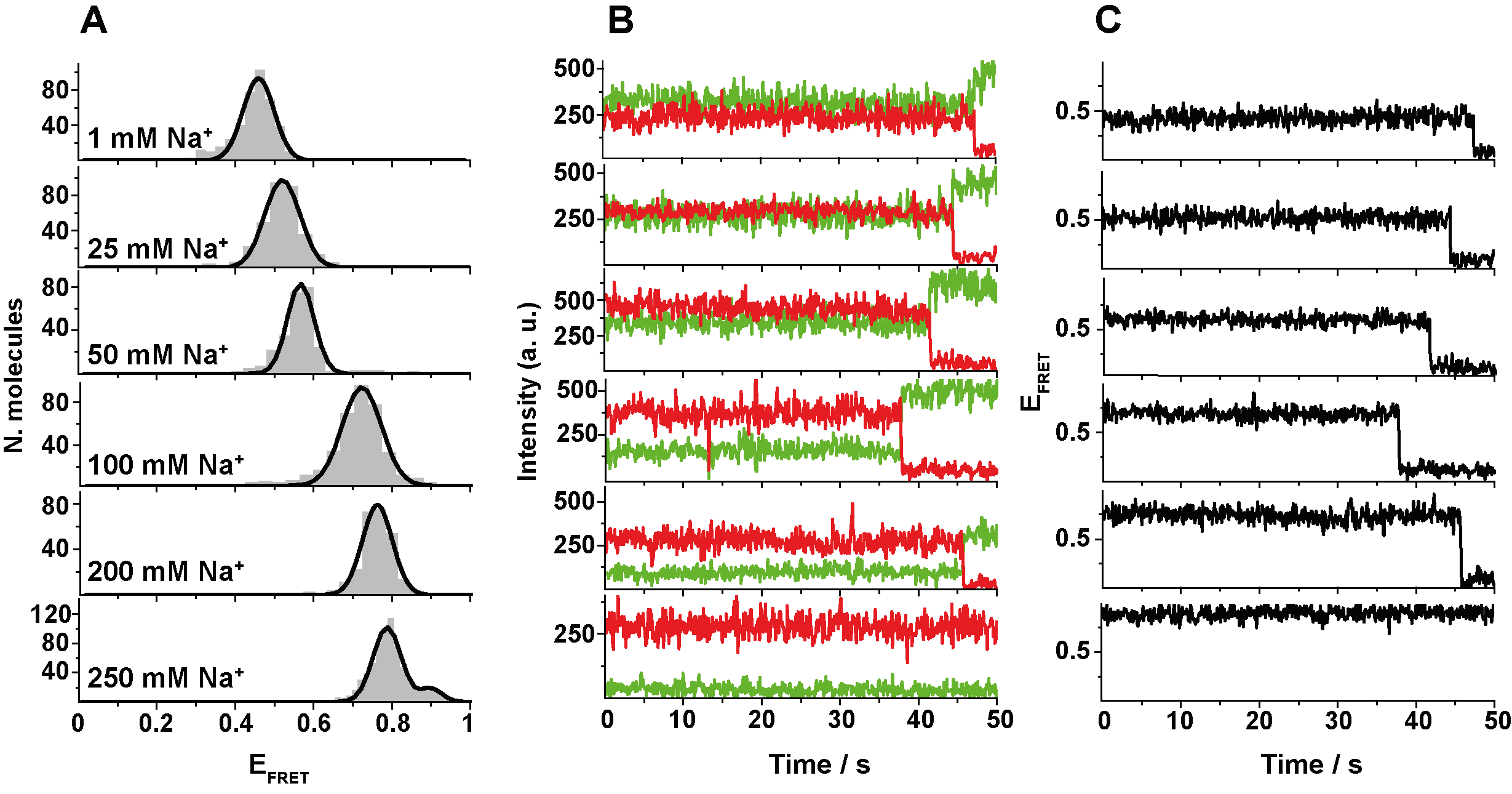


**Supplementary Figure 9 Single-molecule FRET characterization of G4_IX_ as a function of Na^+^ ions**

A) Single-molecule FRET histograms for the G4_IX_ sequence as a function of concentration of Na^+^ ions. Solid line represents the fitting to one or two gaussian functions depending on experimental conditions. (B) Representative single-molecule intensity trajectories obtained as a function of Na^+^ concentration. Emission intensities of donor, acceptor are shown in green and red, respectively. C) Corresponding single-molecule FRET trace extracted from the intensity trajectories shown in B.


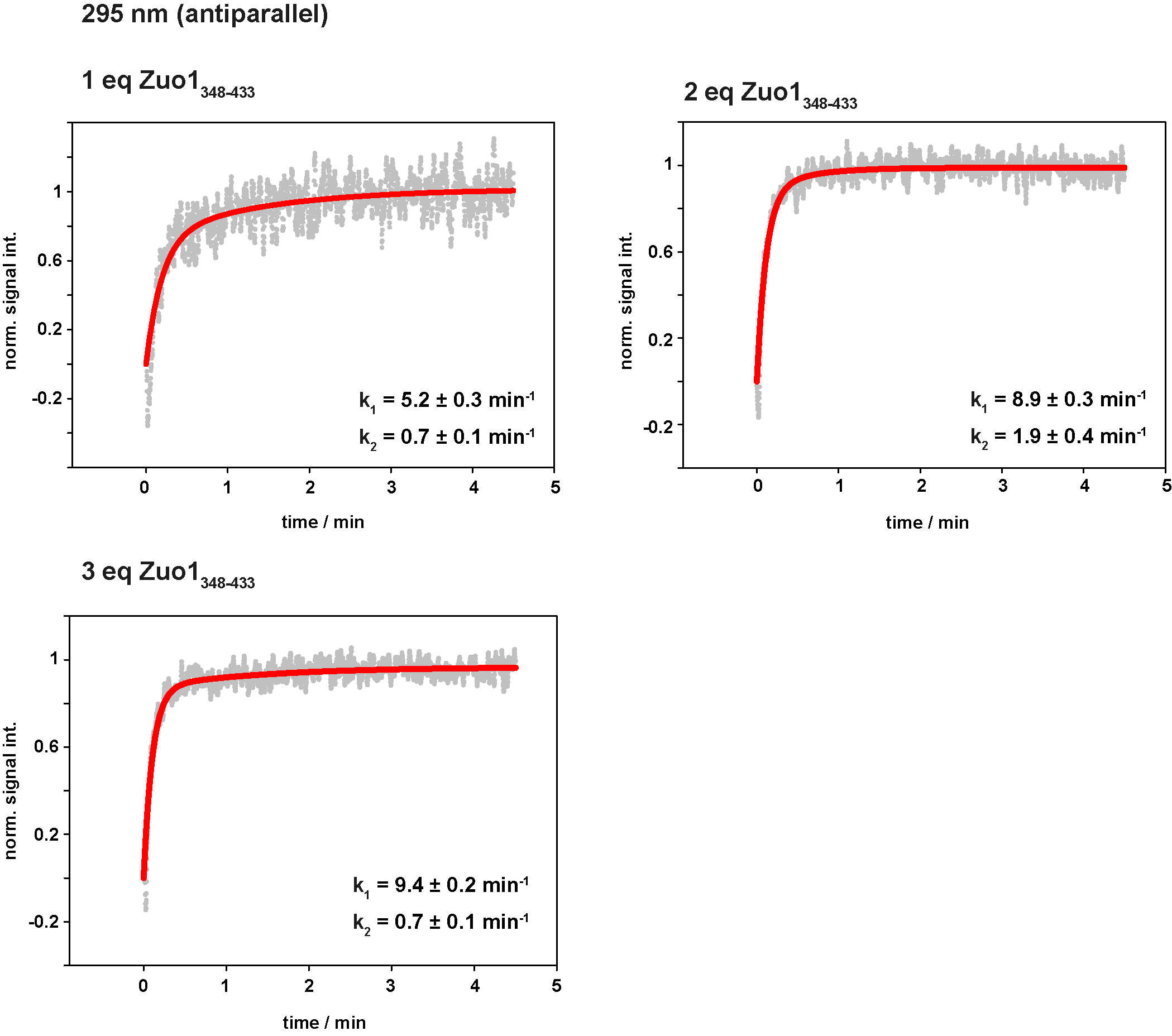


**Supplementary Figure 10 Binding of Zuo1_348-433_ to G4_IX_ is a biphasic process with 1-3 equivalents.**

Kinetics of the stabilization of the antiparallel (295 nm) G4_IX_ signal in the presence of K^+^ after addition of 1, 2 or 3 equivalents Zuo1_348-433_. Kinetic rate constants were taken from the normalized CD signal values. Data have been fitted by bi-exponential regression.

**Supplementary Table 1:** The table shows yeast strains used in this paper. Strains were created by MYC tagging/deleletion/insertion of plasmids in standard laboratory W303 strains.

| **Strain** | **Name** | **Genotype** | **Source** |
| --- | --- | --- | --- |
| 1 | Wildtype (WT) | W303 MATa | R. Rothstein |
| 2 | Zuo1-MYC | MATa; Zuo1-myc trp | De Magis et al., NatComm, 2020 |
| 3 | *zuo1Δ* | MATa; zuo1::kan | This paper |
| 4 | WT+ Zuo1-OE | MATa; pBG1805 Zuo1-OE ura | De Magis et al., NatComm, 2020 |
| 5 | WT + Zuo1_348-433_-OE | MATa; pBG1805 Zuo1_348-433_-OE ura | This paper |
| 6 | WT + scramble | MATa; pBG1805 ura | This paper |
| 7 | *zuo1Δ* + Zuo1-OE | MATa; zuo1::kan pBG1805 Zuo1-OE ura | This paper |
| 8 | *zuo1Δ* + Zuo1_348-433_-OE | MATa; zuo1::kan pBG1805 Zuo1_348-433_-OE ura | This paper |
| 9 | *zuo1Δ* + scramble | MATa; zuo1::kan pBG1805 ura | This paper |

**Supplementary Table 2:** Used oligonucleotides for biophysical studies of selected G4 motifs (with two, three or four G-tracts) on different chromosomes.

| **Name** | **Sequence** |
| --- | --- |
| G4_Ib_ | GGTGGAAGGGACAAGG |
| G4_XIV_ | GGAAGGTGGATCAGG |
| G4_XII_ | GGATGGGACGGCGG |
| G4_XI_ | GGGGAGGGGAAAAGAAAAGGGGAAGGGG |
| G4_IV_ | GGGGAGGGGAAGGGGAGGGG |
| G4_III_ | GGGCGGGCCTTGCCATAGGGCCACGGG |
| G4_IX_ | GGGTACGGTGGGTAATAAGGGAAGGTATCGGG |

**Supplementary Table 3:** Primers listed which were used for qPCR analysis after ChIP experiments. The primers amplify regions with selected G4 motifs (with two, three or four G-tracts) on different chromosomes including one control without any G4 motif.

| **Name** | **Location** | **Orientation** | **Sequence** |
| --- | --- | --- | --- |
| G4 tract2 Ib | Chr. Ib | Fw | CCGATCCAGTCCCACAGTAA |
|  |  | Rv | TGATCCTTGTCCCTTCCACC |
| G4 tract2 XIV | Chr. XIV | Fw | CGGGCGCCAAATCAATCATA |
|  |  | Rv | TTTGTTCCGGACGTGATTGC |
| G4 tract2 XII | Chr. XII | Fw | TCCCATTCACCTATTCTGGCA |
|  |  | Rv | TTCGCAATCACACTTCATGC |
| G4 tract3 IX | Chr. IX | Fw | TGTAGAGTCTTTGGCACTGTTG |
|  |  | Rv | GGCATTACAACTCGCAAAACC |
| G4 tract3 III | Chr. III | Fw | ATTGCACAGCCATTGGATCG |
|  |  | Rv | CATGTGTGGTGTGGTTTGGA |
| G4 tract4 XI | Chr. XI | Fw | CGATGTAAGGCACCCAGG |
|  |  | Rv | TCCTAGTGCTGGCAAAATGA |
| G4 tract4 IV | Chr. IV | Fw | AGTTATGTTTGTAGCCGGCG |
|  |  | Rv | AAGGAAGGAATTTGGGTCTCAA |
| No G4 VI | Chr. VI | Fw | AGCAGAATAATCCGGAGCTGA |
|  |  | Rv | ACTACTCAATTGTTTTCCATGGG |

**References**

[1] J. K. Ducett, F. C. Peterson, L. A. Hoover, A. J. Prunuske, B. F. Volkman, E. A. Craig, Unfolding of the C-Terminal Domain of the J-Protein Zuo1 Releases Autoinhibition and Activates Pdr1-Dependent Transcription, J. Mol. Biol. **2013**, *425*, 19–31.
